# Roast: a tool for reference-free optimization of supertranscriptome assemblies

**DOI:** 10.1186/s12859-023-05614-4

**Published:** 2024-01-02

**Authors:** Madiha Shabbir, Aziz Mithani

**Affiliations:** Department of Life Sciences, Syed Babar Ali School of Science and Engineering, Lahore University of Management Sciences (LUMS), DHA, Lahore, 54792 Pakistan

**Keywords:** Assembly improvement, Supertranscriptome assembly, Reference-free optimization, Assembly errors, RNA-seq, Supertranscript

## Abstract

**Background:**

Transcriptomic studies involving organisms for which reference genomes are not available typically start by generating de novo transcriptome or supertranscriptome assembly from the raw RNA-seq reads. Assembling a supertranscriptome is, however, a challenging task due to significantly varying abundance of mRNA transcripts, alternative splicing, and sequencing errors. As a result, popular de novo supertranscriptome assembly tools generate assemblies containing contigs that are partially-assembled, fragmented, false chimeras or have local mis-assemblies leading to decreased assembly accuracy. Commonly available tools for assembly improvement rely primarily on running BLAST using closely related species making their accuracy and reliability conditioned on the availability of the data for closely related organisms.

**Results:**

We present ROAST, a tool for optimization of supertranscriptome assemblies that uses paired-end RNA-seq data from Illumina sequencing platform to iteratively identify and fix assembly errors solely using the error signatures generated by RNA-seq alignment tools including soft-clips, unexpected expression coverage, and reads with mates unmapped or mapped on a different contig to identify and fix various supertranscriptome assembly errors without performing BLAST searches against other organisms. Evaluation results using simulated as well as real datasets show that ROAST significantly improves assembly quality by identifying and fixing various assembly errors.

**Conclusion:**

ROAST provides a reference-free approach to optimizing supertranscriptome assemblies highlighting its utility in refining de novo supertranscriptome assemblies of non-model organisms.

**Supplementary Information:**

The online version contains supplementary material available at 10.1186/s12859-023-05614-4.

## Background

With rapid advances in sequencing techniques, RNA sequencing, or RNA-seq, has emerged as a technique of choice for the characterization and comparison of transcriptome at a genome-wide level. Studies performing various transcriptomic analyses such as expression profiling of genes, variant analysis, novel transcripts identification or fusion gene detection typically start by mapping RNA-seq reads back to the reference genome or transcriptome [1, 2]. In cases where a reference genome or transcriptome is not available, which is typically the case for non-model organisms, studies typically start by generating a *de novo* transcriptome assembly for the organism under study. For this, raw RNA-seq reads are assembled into contigs, corresponding to expressed transcripts, using one of the several popular reference-free transcriptome assemblers such as Trinity [3], Oases [4], Trans-ABySS [5], IDBA-tran [6], and SOAPdenovo-Trans [7]. More recently, studies involving RNA-seq data have started using supertranscritome references instead of transcriptome references [8–11]. A supertranscriptome is a type of transcriptome reference which combines all transcribed splice variants for a gene in one supertranscript thereby providing a compact reference for read alignment and subsequent downstream analyses [8, 12].

**Fig. 1:**
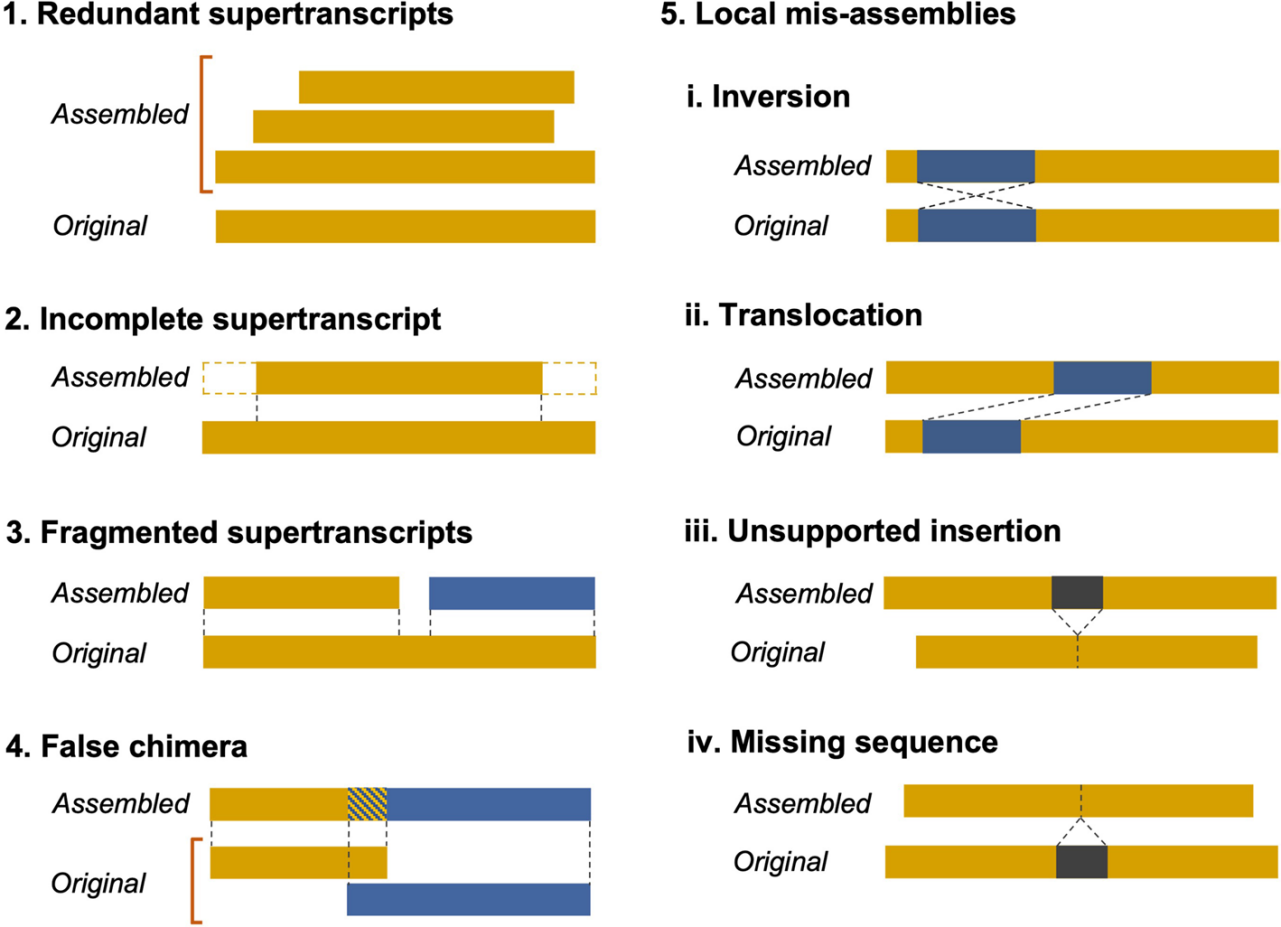
Common errors in de novo supertranscriptome assemblies. Redundant transcripts occur when multiple copies of the same transcripts are generated. Incomplete supertranscripts occurs when one or more exons are fully or partially missing either at one side or both side of assembled contigs. Fragmented supertranscripts correspond to two or more contigs relating to different regions of a supertranscript. False chimeras are contigs generated as a result of erroneous fusion of two or more full or partial supertranscripts. Local mis-assemblies are characterized as missing sequences, unsupported insertions, inversions and/or translocations in the contigs

Assembling a transcriptome or supertranscriptome using RNA-seq reads is, however, a challenging task due to significantly varying abundance of mRNA transcripts, alternative splicing, gene duplication and sequencing of intronic regions present in pre-mRNA or decaying of mature mRNA [13–16]. The problem is further compounded by the presence of sequencing errors in the underlying data and the computational limitations of the algorithms used by these assemblers, which typically adopt a number of heuristics to speed up the assembly process [17–19]. As a result, while the assemblers produce a workable assembly, they also generate a number of erroneous contigs which do not truly represent underlying biological supertranscripts thereby decreasing the overall assembly accuracy [18, 20]. Some of the common supertranscriptome assembly errors are shown in Fig. 1. These include (i) supertranscript redundancy whereby multiple copies of the same supertranscript are generated due to underlying DNA polymorphism or sequencing errors, (ii) incomplete supertranscript where one or more whole or partial exons are missing either at one side or both sides of the assembled contig, (iii) fragmented supertranscript where two or more contigs corresponding to different regions of a supertranscript are present which could not be joined together during the assembly process, (iv) false chimeras which correspond to contigs generated as a result of erroneous fusion of two or more full or partial supertranscripts, and (v) local mis-assemblies and errors which are characterized as missing sequences, unsupported insertions, inversions and/or translocations in the contigs [18, 21–24]. These assembly errors not only prevent accurate functional annotation of the supetranscriptome but also affect the downstream analyses such as identification of differential gene expression, splice variants and homologous genes [24].

In recent years, a few tools have emerged to correct one or more of the aforementioned assembly errors. For example, DRAP fixes partial contigs by re-assembling them using RNA-seq data and evaluates the results by comparing against a user-provided reference protein sequences [13]. Similarly, BRANCH identifies and completes partial contigs by taking information from genomic assembly along with transcriptome assembly and RNA-seq reads to extend incomplete contigs and obtain full-length transcripts from partial contigs [16]. However, as reported by the authors themselves, this approach may negatively affect the transcriptome assembly improvement due to errors in the genomic assembly itself [16]. To deal with false chimeras, the most commonly used approach is the comparison of assembly contigs with closely related species using BLAST [24] making the reliability of the results conditioned on the availability and quality of the data for closely related organisms. DRAP [13], on the other hand, checks for the presence of one unique full-length open reading frame (ORF) per transcript to identify and split chimeric contigs. While this seems a very good approach to distinguish normal contigs from chimeras, however, in practice it is not viable due to the presence of incomplete and/or fragmented contigs and chimeras resulting from the fusion of partial contigs. Moreover, ORFs are not defined for supertranscripts making this approach unfeasible when working with supertranscriptomes. None of the above-mentioned tools addresses the issues of local mis-assemblies. Also, to the best of our knowledge, no tool attempts to correct any of these assembly errors in a supertranscriptome.

Here, we present ROAST: Reference-free Optimization of Assembled SuperTranscriptomes, a tool which aims to simultaneously correct all the assembly errors highlighted above and enables reference-free optimization of supertranscriptome assemblies. ROAST is an iterative tool, which uses paired-end information of the reads produced from Illumina sequencing platform and error signatures including soft-clips, unexpected expression coverage, and reads with mates unmapped or mapped on a different contig [22] generated by RNA-seq alignment tools to identify and fix supertranscriptome assembly errors. We demonstrate ROAST by generating and improving *de novo* supertranscriptome assemblies of five model organisms from previous analyses [7, 23] including human (*Homo sapiens*), mouse (*Mus musculus*), chicken (*Gallus gallus*), rice (*Oryza sativa*) and arabidopsis (*Arabidopsis thaliana*) as well as the assemblies generated using synthetic paired-end read data simulated from all these species.

## Results and discussion

### ROAST overview

**Fig. 2:**
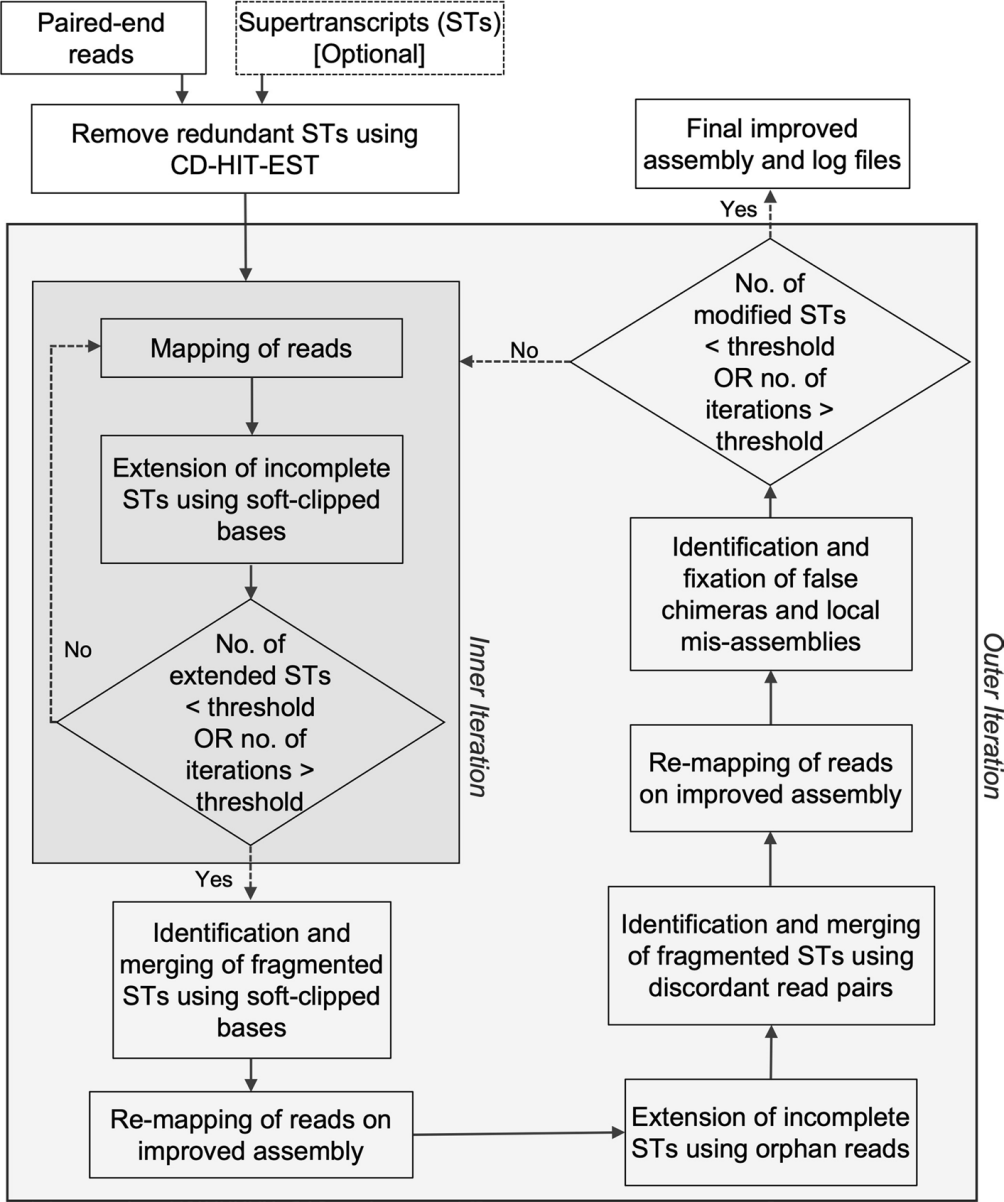
ROAST workflow to identify and fix de novo supertranscriptome assembly errors using RNA-seq data. ROAST takes paired-end RNA-seq reads and the *de novo* supertranscriptome assembly (optional) as input. It starts by removing the redundant supertranscripts and subsequently performs assembly improvement as two nested iterations. At the start of each outer iteration, an inner iteration is run that extends incomplete supertranscripts using soft-clipped bases. The inner iteration starts by mapping the reads on to the assembly from which partially mapped reads (reads containing soft-clipped bases) are extracted and used to extend incomplete contigs. This is done until the number of iterations or the number of contigs containing partially mapped reads reach the user-defined threshold. Once out of the inner iteration, ROAST merges fragmented supertranscripts using partially mapped reads. This is followed by realignment of reads on the improved assembly, which is then used to extend partial supertranscripts and merge fragmented contigs using reads with unmapped mates (orphan reads) and discordantly mapped read pairs respectively. The resulting assembly is then used for re-mapping of reads and subsequently false chimera and local mis-assemblies are identified and fixed. This whole process is repeated until the number of iteration or the number of contigs containing errors reach the user-defined threshold. At the end of iterative improvement, ROAST provides final improved assembly as output

ROAST is a command line tool which provides reference-free improvement of supertranscriptome assemblies by fixing different assembly errors (Fig. 1) using RNA-seq data without relying on BLAST searches against other organisms. ROAST uses Illumina paired-end sequencing data for assembly improvement since it is the method of choice for majority of the studies involving *de novo* supertranscriptomic assembly [25]. ROAST takes paired-end RNA-seq data and the transcriptome assembly to be optimized in the form of supertranscripts [8] generated from these reads as input (Fig. 2). If the assembly is not provided then ROAST generates the supertranscriptome assembly using Trinity assembler [3].

ROAST begins assembly improvement process by removing redundant contigs present in the supertranscriptome assembly using CD-HIT-EST [26] (see “Removal of redundant contigs” Section in the Methods below) since tools like Trinity and Trans-ABySS have been reported to generate redundant contigs [9, 21, 27]. Once redundancies have been removed from the assembly, ROAST aligns the RNA-seq reads to the assemblye “Removal of redundant contigs” (see the "Methods" Section) and rigorously processes this alignment data in an iterative manner to investigate different error signatures such as discordantly-mapped reads (reads with mates unmapped or mapped on a different contig), partially mapped reads (reads containing soft-clipped bases) and unexpected variation in the read coverage along the contig to identify and fix different assembly errors.

Assembly refinement in ROAST runs as two nested iterations, which we refer to as inner and outer iterations (Fig. 2). At the beginning of each outer iteration, first an inner iteration is run that extends partially assembled contigs. At each step of the inner iteration, consensus sequences are generated using the soft-clipped bases from the reads that map near the ends of the contigs to extend contig sequences (see the  “Extending incomplete supertranscripts” Section below). This is done until the number of iteration reaches a user-defined threshold (default value: 30) or no further improvement is observed. Once the inner iteration is completed, ROAST performs BLAST [28] searches using blastn algorithm within the assembly to identify potential overlap between different contigs. Contigs containing significant overlaps are merged together to form longer contigs (see the “Merging fragmented supertranscripts” Section below). ROAST uses this improved assembly to further extend partially assembled contigs and merge fragmented contigs using reads with unmapped mates (see the  “Extending incomplete supertranscripts” Section below) or distantly mapped mates (see the “Merging fragmented supertranscripts” Section below). This is followed by splitting of false chimeras (see the “Splitting false chimeras” Section below) and fixing of local mis-assemblies (see the “Fixing local mis-assemblies” Section below) by exploiting read coverage and soft-clipped bases in partially mapped reads (Fig. 2). The outer iteration is repeated until the number of iteration reaches a user-defined threshold (default value: 100) or the number of contigs containing errors reach a user-defined threshold (default value: 0). By using a nested iterative approach to systematically identify and fix assembly errors, ROAST produces an assembly which is significantly improved compared to the initial assembly without the need of a reference genome or transcriptome.

Once the improvement process is over, ROAST writes the following files as output: a FASTA file containing the improved assembly, a summary file showing the mapping between the contigs present in initial and final assemblies, intermediate assemblies and log files detailing the changes made at each step of the iterative improvement process to help users inspect the errors present in the assembly and monitor the changes made during the iterative improvement process.

### ROAST algorithm

**Fig. 3:**
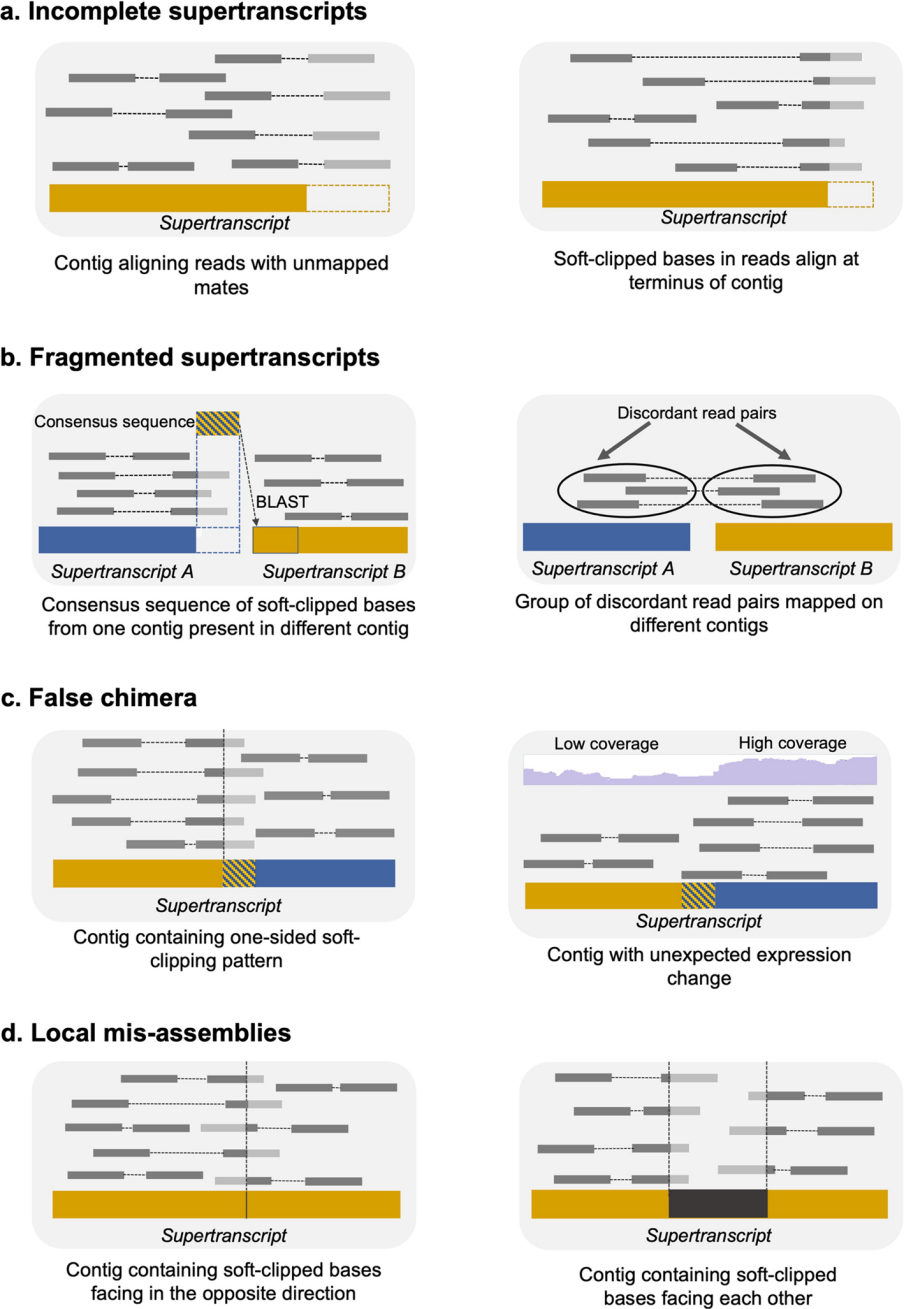
De novo supertranscriptome assembly error signatures used by ROAST to identify various types of assembly errors. **a** Incompleteness of the supertranscripts is detected using unmapped or partially reads (reads containing soft-clipped bases) at the edges of the contig. **b** Fragmented contigs are identified using partially mapped reads such that the soft-clipped bases map on different contigs and using reads with mates mapped on different contigs. **c** False chimeras are identified using partially mapped reads occurring inside a contig as well as based on unusual change in expression level along a contig. **d** Local mis-assemblies can be detected using partially mapped reads with soft-clipped bases occurring in either a crisscross fashion (missing sequences, inversions and translocations) or facing towards each other (unsupported insertions)

ROAST is an iterative tool that identifies and fixes various supertranscriptome assembly errors (Fig. 1) using different error signatures such as soft-clips, unexpected change in read coverage, and discordantly mapped reads generated during read alignment. The algorithm is summarized in Fig. 3 and described in detail for different error types in the subsequent subsections.

#### Extending incomplete supertranscripts

Incomplete supertranscripts are one of the most common supertranscriptome assembly errors. They correspond to contigs where one or more whole or partial exons are missing either at one side or both sides of the contigs resulting in missing sequence (Fig. 1). When working with supertranscriptome, they correspond to contigs that do not represent full-length supertranscripts. To identify and fix such contigs, ROAST uses partially mapped reads found near the edges of a contig and reads with unmapped mates as described below.

##### Reads partially mapped at the edges of a contig

Partially mapped reads are the reads containing soft-clipped bases. Soft-clipped bases are the unmatched portion of an aligned read that do not support the nucleotides of the corresponding contig and are, therefore, masked during alignment [29]. This primarily occurs when these unmapped portions either do not map anywhere in the assembly due to missing reference sequence (incomplete transcript) or map on a different contig due to the presence of a fragmented transcript (see the “Merging fragmented supertranscripts” Section below) or an incorrectly assembled sequence (see the “Fixing local mis-assemblies” Section below) [22]. To extend incomplete sequences, ROAST first identifies reads containing soft-clipped bases in the outward direction that occur within 25 bases (user-defined parameter; default value: 25) of the contig boundary (Additional file 1: Figs. S1 and S2). Soft-clipped bases present in at least 3 mapped reads (user-defined parameter; default value: 3) and supported by at least 75% of the mapped reads (user-defined parameter; default value: 75) are then used to generate consensus sequence(s) using CAP3. If CAP3 fails to generate a consensus sequence, for example in the case for short sequences, ROAST generates the consensus sequence by assigning the base occurring with the highest frequency in the soft clipped bases at each position. If two or more bases have maximum coverage at a position, ROAST uses the corresponding IUPAC code to represent those bases at that position. The consensus sequence(s) is then used to extend incomplete corner(s) of the contig. The extension is done inside the inner iteration until the number of iterations reach a user-defined threshold or no further improvement is observed (see Fig. 2 and the “ROAST overview” Section above). To reduce time and memory taken by re-alignment of reads in each iteration, ROAST uses only those reads for re-alignment that are mapped at contig edges, that is within certain bases from the contig boundaries (default value: 2 $\times$ read length) in the current iteration.

##### Reads with unmapped mates

Besides partially mapped reads, another signature for an incomplete supertranscript is a cluster of reads mapped at the edge(s) of a contig with unmapped mates [22]. Presence of such reads suggests that the sequence corresponding to their unmapped mates is missing from the assembly. ROAST exploits this information by identifying reads with unmapped mates, which are mapped in the outward direction at the contig edges (see the Paragraph “Reads partially mapped at the edges of a contig” in this Section). To reduce false positives, at least 3 reads with unmapped mates (user-defined parameter; default value: 3) are required. In addition, ROAST extracts unmapped mates only for those reads that have less than 25% (user-defined parameter; default value: 25) soft-clipped bases. The unmapped mates are then re-assembled using CAP3 [30] and the contig is extended by stitching it to the newly assembled sequence based on the overlapping edges (Additional file 1: Figs. S3 and S4). Only those extensions are considered valid which increase the contig length by at least 50% of the read length (user-defined parameter; default value: 50). If no overlap is found then the newly assembled sequence is tagged and added to the assembly as a separate contig. If the newly assembled sequence is not merged with the original contig by the end of the iterative improvement due to lack of overlap, it is joined to the contig with 5 Ns (user-defined parameter, default value: 5).

#### Merging fragmented supertranscripts

Another common supertranscriptome assembly error relates to fragmented supertranscripts. A fragmented supertranscript refers to a set of two or more contigs in the assembly which belongs to a full-length supertranscript but could not be joined together during the assembly process. ROAST uses partially mapped reads with soft-clipped bases mapping on different contigs and discordantly mapped read pairs to identify and fix fragmented supertranscripts as described below.

##### Partially mapped reads with soft-clipped bases mapping on different contigs

As noted in the  “Extending incomplete supertranscripts” Section above, soft-clipped bases from partially mapped reads can be used to identify fragment supertranscripts in addition to other assembly errors. If the consensus sequence generated from the soft-clipped bases from the reads partially mapped near the end of a contig maps on a different contig, this suggests that the two contigs belong to one full-length transcript which could not be assembled together due to insufficient coverage depth and/or assembly error. ROAST, immediately after exiting the inner iteration, processes the contigs that have been extended using soft-clips during the inner iteration to identify and merge fragment supertranscripts. (see Fig. 2). This is done as follows. First, ROAST uses 25 bases (user-defined parameter; default value: 25) as initial query sequence to search for an overlap across the whole assembly using BLAST (blastn algorithm with default parameters). A hit with 100% identity (user-defined parameter; default value: 100) with the initial query sequence is taken forward. In case of multiple hits, the hit with the maximum score ((identity/query length) * 100) is considered. Subsequently, the overlapping region between the original contig and the contig identified by BLAST is extended, if possible, using a relaxed criteria of 90% identity (user-defined parameter; default value: 90). By default, hits containing gaps are ignored when searching for overlapping sequence although this behaviour can be changed by the user by setting the ‘number of gaps allowed in a hit’ parameter (default value: 0). Once the maximum overlap is found, the two contigs are merged to create a longer contig (Additional file 1: Fig. S1).

##### Reads with mates mapped on a different contig

A read and its mate are expected to map on the same contig during the alignment process since they belong to the same physical transcript molecule. ROAST exploits this fact to identify fragmented supertranscripts by looking for a cluster of reads aligned near the contig edges (see the “Extending incomplete supertranscripts” Section above) with mates mapped on a different contig. A cluster of such reads is referred to as a read or mate island in ROAST. A similar strategy has been used by Grouper to cluster similar contigs together [31]. To avoid false positives due to alignment errors, ROAST requires at least 5 reads (user-defined parameter) to be present in a read/mate island. To merge parts of a fragmented supertranscript, contigs containing read and mate islands are first identified. These contigs are then merged based on their overlapping edges to create a longer contig (Additional file 1: Figs. S5 and S6). An overlap is only deemed valid for merger if the overlap length between contigs is 10 or more bases (user-defined parameter; default value: 10), BLAST score is 90% or more (user-defined parameter; default value: 90) and the overlapping region starts within a certain bases from the outer edge of the read/mate island (default value: 5% of the read length).

#### Splitting false chimeras

False chimera is another type of assembly error which significantly reduces assembly quality. It corresponds to a contig generated as a result of erroneous fusion of two or more full or partial supertranscripts [24] and is different from rarely existing natural chimeric transcripts (fusion genes) in some cancer tissues [32, 33]. False chimera can be “self-chimera”, where full or a part of supertranscript is duplicated and fused to itself or “multi-supertranscript chimera”, which is generated by the fusion of multiple supertranscripts [24]. ROAST removes repeated segments of self chimeras and splits multi-supertranscript chimeras using the following strategies.

##### Reads partially mapped inside a contig

While reads partially mapped at the edge of a contig provide signatures for incomplete and fragmented contigs (see the  “Extending incomplete supertranscripts” and “Merging fragmented supertranscripts” Sections above), their occurrence in the middle of a contig suggests the presence of false chimera or local mis-assemblies (see “Fixing local mis-assemblies” Section below). To identify false chimeras, ROAST looks for reads partially mapped inside a contig such that soft-clipped bases occur in only one direction (see Fig. 3 and Additional file 1: Fig. S7). While searching for soft-clips, it ignores the soft-clipped bases occurring at the exon-exon boundaries (see the “Methods” Section) since they can also lead to partial mapping of reads [34]. Furthermore, ROAST only considers positions containing soft-clips which are supported by at least 75% of the mapped reads (user-defined parameter; default value: 75) and the consensus sequence resulting from these soft-clipped bases (see the Paragraph “Reads partially mapped at the edges of a contig” above) contains at least 10 bases (user-defined parameter; defualt value: 10). It then uses BLAST (blastn algorithm with default parameters) to check if the consensus sequence occurs anywhere else in the contig with an identity of $\ge$ 90% (user-defined parameter; default value: 90). If found, the region between the BLAST hit and the position containing soft-clipped bases is extracted and the flanking regions are joined together using overlap between their bases (see Additional file 1: Fig. S7). In the case of no hit, the contig is split into two at the soft-clip position and the smaller sequence is extracted. To distinguish between self and multi-supertranscript chimeras, the extracted sequence is searched against the resulting contig again to check for duplication. A BLAST score of $\ge$ 90% (user-defined parameter; default value: 90) is required for the sequence to be regarded as self-chimera and consequently removed from the assembly. If no significant hit is found then depending on whether the sequence length is $\ge$ 200 bases (user-defined parameter; default value: 200) either a new contig is created from the sequence or it is removed from the assembly thereby preventing unnecessary addition of fragmented contigs in the assembly (see Additional file 1: Figs. S7, S8 and S9).

##### Unusual changes in the transcript expression levels

A typical mapping of RNA-seq data results in a consistent read coverage with random fluctuations across the length of the transcripts. However, an abrupt increase or decrease of coverage or a gradual but abnormal coverage change suggests the presence of a false chimera [22]. For multi-transcript chimera, the change in the read coverage can be attributed to the difference in expression levels of the different genes whereas in the case of self-chimera this occurs due to the reads mapping on multiple (original and duplicated) locations. ROAST detects such unusual changes in the expression level to identify false chimeras as follows. First, the contig is scanned from left to right to detect abrupt coverage changes. A difference of $\ge$80% in read coverage (user-defined parameter; default value: 80) between two consecutive positions is regarded as an abrupt change. If a chimeric position is identified, the contig is split into two at that position and the smaller sequence is extracted. This sequence is either removed from the assembly or added as a new contig depending on its length and the BLAST result, as described above (see the Paragraph “Reads partially mapped inside a contig” in this Section) (Additional file 1: Fig. S10). Since the RNA-seq alignment typically results in a steady decrease in the coverage towards the ends of a contig [35], ROAST ignores coverage changes within certain bases from the ends of the contig (default value: read length) while looking for chimeric positions. User can optionally search for gradual but abnormal coverage changes using a sliding window approach, where average read coverage for two consecutive windows of size 100bp (user-defined parameter) each flanking a position is calculated and compared. A difference of $\ge$80% (user-defined parameter; default value: 80) between the average coverage of the two windows is required for a position to be regarded as chimeric. While this approach can detect genuine false chimeras (Additional file 1: Figs. S11 and S12), the presence of fluctuation in the read coverage may lead to false positives and is, therefore, off by default in ROAST.

#### Fixing local mis-assemblies

Local mis-assemblies in the de novo assembled contigs are characterized as structural errors such as inversions and/or translocations, unsupported insertions, and missing sequences. These structural abnormalities can be detected using reads partially mapped inside contigs depending on how soft-clipped bases appear in these reads. To identify and fix local mis-assemblies, ROAST scans each contig from left to right and looks for different patterns of the soft-clip bases as described below.

##### Soft-clipped bases facing in the opposite direction

Crisscrossed soft-clipped bases or soft-clipped bases facing in the opposite direction occurring at a specific position inside a contig can help to identify and complete missing sequences in the assembled transcripts/supertranscripts in addition to providing signatures for inversion and translocation (see Additional file 1: Fig. S13). To identify these mis-assemblies, consensus sequences from the crisscrossed soft-clipped bases at non exon-exon boundary positions are searched against the contig using BLAST (blastn algorithm with default parameters) using the same strategy as described before (see the “Splitting false chimeras” Section). If no hit is found for both the consensus sequences then the sequences are flagged as missing from the contig, which need to be added to the current contig. Based on whether overlapping bases are found between 5’-end of left soft-clip consensus sequence and 3’-end of the right soft-clip consensus sequence, the two sequences are either merged or concatenated end-to-end and the resulting sequence is inserted in the contig at the position containing soft-clipped bases (Additional file 1: Fig. S14). If, on the other hand, one or both consensus sequences are found anywhere else in the contig, the corresponding region(s) is flagged as translocation and optionally, inversion depending on the orientation of the BLAST hit. To fix such cases, ROAST extracts and inserts the translocated/inverted fragment(s) at the correct position(s) in the appropriate direction(s) (see Additional file 1: Fig. S15).

##### Soft-clipped bases facing each other

Like crisscrossed soft-clipped bases, soft-clipped bases facing towards each other can also help to identify local mis-assemblies. Specifically, they provide signature for unsupported insertions. An unsupported insertion corresponds to bases in the contigs that are not supported by read evidence [22] and, therefore, allows only partial mapping of reads around it (Additional file 1: Fig. S16). The distance between the two positions containing soft-clipped bases equals to the size of inserted fragment. To identify unsupported insertions, ROAST identifies soft-clip positions which are facing each other using the same strategy as described before (see the “Splitting false chimeras” Section). For a region to be flagged as an unsupported insertion, the left consensus sequence (see the Paragraph “Reads partially mapped at the edges of a contig” above) must map immediately after the position generating right consensus sequence and vice versa (Additional file 1: Fig. S17). Regions flagged as unsupported insertions which are less than 200 bases (user-defined parameter; default value: 200) are discarded while longer fragments are checked for false chimera (see the “Splitting false chimeras” Section above).

### Evaluation of ROAST’s performance

We evaluated the performance of ROAST using benchmark datasets based on simulated as well as actual sequencing data. The evaluation results are discussed below.

#### Evaluation using simulated datasets

To assess the accuracy of ROAST in identifying different types of assembly errors, we constructed reference supertranscriptomes for human (*Homo sapien*), mouse (*Mus musculus*), chicken (*Gallus gallus*), rice (*Oryza sativa*) and Arabidopsis (*Arabidopsis thaliana*) using their publicly available reference genomes (see the “Construction of reference supertranscriptomes” Section in Methods). We produced 800 simulated errors, 200 for each error type (incomplete supertranscripts, fragmented supertranscripts, false chimeras and local mis-assemblies) in each reference supertranscriptome (see Section “Simulated assembly errors” in Methods). We further generated 40 million simulated read pairs for each species (see the Section “Simulated RNA-seq data” in Methods) to detect these errors. Simulated RNA-seq data provides true representation of the real supertranscripts obtained from the reference sequences of model organisms and can, therefore, be used to assess the accuracy of error identification and fixation in the reference supertranscriptomes [22, 36].

ROAST was run to identify and correct errors produced in supertranscriptomes of model organisms using the simulated reads with default parameters with two exceptions. First, check for duplicate supertranscript was turned off by setting “–cdhitest 0” since supertranscriptome references were manually created using their respective transcriptome assemblies downloaded from public databases and were not expected to contain duplication. Second, check for exon-exon boundary when processing soft-clipped basses was disabled since simulated reads were produced directly from supertranscripts and hence did not contain soft-clipped bases at the exon-exon boundaries.

Table 1 shows the number of errors identified and fixed for each error type in different species. Incomplete supertranscripts and missing sequences were regarded as correctly fixed if $>=90\%$ of the deleted sequence was recovered. A complete recovery of the original sequence was required for fragmented supertranscripts, translocations and inversions. Similarly, unsupported insertions was regarded as properly fixed if all extra bases were removed. For false chimeras, both sequences producing a chimeric contig were required to be fully restored for the error to be deemed as rectified. It can be seen that ROAST identified and fixed assembly errors that were simulated in the reference supertranscriptomes with high accuracy. ROAST was most accurate in identifying and correctly recovering missing sequences and performed with 100% accuracy in four out of five species. It also fixed other local mis-assemblies and merged fragmented contigs with almost 100% accuracy across all species and was able to recover incomplete sequences with $90\%$ or more accuracy in three out of five species. For false chimeras, ROAST was able to identify and correct between 83.5% and 88% errors that were produced in the reference supertranscriptome assemblies.

**Table 1:** ROAST performance in identifying and fixing different types of simulated errors using simulated RNA-seq data

Organism	Incomplete supertranscript	Fragmented supertranscript	False chimera	Local mis-assembly			
				Missing sequence	Unsupported insertion	Trans-location	Inversion
Human	174 (87)	194 (97)	174 (87)	48 (96)	50 (100)	49 (98)	49 (98)
Mouse	172 (86)	196 (98)	167 (84)	50 (100)	49 (98)	48 (96)	49 (98)
Chicken	179 (90)	196 (98)	176 (88)	50 (100)	49 (98)	49 (98)	46 (92)
Rice	193 (97)	196 (98)	168 (84)	50 (100)	50 (100)	46 (92)	48 (96)
Arabidopsis	192 (96)	192 (96)	169 (85)	50 (100)	49 (98)	46 (92)	49 (98)

Percentages are shown in parentheses. A total of 200 errors were produced for each error type including partial and fragmented supertranscripts and false chimeras. For local mis-assemblies, 50 errors were produced for each error type

To further see how well ROAST optimization led to the restoration of original assembly quality, we compared the TransRate score of the initial, erroneous and the ROAST-improved assemblies. TransRate score is widely used as a key indicator of assembly quality and takes into account various factors including nucleotide identity, number and order of nucleotides in the contig along with the probability of univariate coverage depth for a contig calculated from aligned reads [22, 25]. The results for the comparison are given in Table 2. As expected, erroneous assemblies had slightly lower TransRate scores due to the presence of simulated assembly errors. By removing these assembly errors and restoring the assemblies close to their original state, ROAST was able to restore the scores to the initial levels. Taken together, these results indicate that ROAST is able to correctly identify and fix different types of supertranscriptome assembly errors with extremely high accuracy across different species.

**Table 2:** Comparison of overall assembly quality before and after the correction of simulated errors using simulated RNA-seq data against the original reference assembly

Organism	Assembly	TransRate score
Human	Reference	0.80
	Erroneous	0.79
	Improved	0.80
Mouse	Reference	0.98
	Erroneous	0.97
	Improved	0.98
Chicken	Reference	0.83
	Erroneous	0.81
	Improved	0.82
Rice	Reference	0.81
	Erroneous	0.79
	Improved	0.81
Arabidopsis	Reference	0.83
	Erroneous	0.81
	Improved	0.83

#### Evaluation using real datasets

The performance of ROAST for de novo supertranscriptome assembly improvement was tested using published data for the five model organisms used in this study. These species have well-annotated reference assemblies and have been used to benchmark performance in previous studies [22, 23, 31]. RNA-seq data was downloaded from NCBI Sequence Read Archive (see the “Real datasets” Section in Methods) and used to generate de novo supertranscriptome assemblies using Trinity assembler (see the section “De novo supertranscriptome assembly of model organisms” in Methods). Next, ROAST was run with default parameters to identify and fix errors present in these assemblies. In all cases, ROAST was run until no further assembly improvement could be made. The numbers of contigs in which different types of errors were identified and fixed by ROAST for each dataset are listed in Additional file 1: Table S3. The improved assemblies were compared with the initial assemblies and the results were evaluated against the reference supertranscriptomes created for these organisms (see the “Construction of reference supertranscriptomes” Section in Methods), which provide a means to objectively evaluate the assembly improvements. We calculated various metrics for initial and improved assemblies using different evaluation tools including TransRate [22], rnaQUAST [37], and Samtools [38]. The results are discussed below.

Completeness of supertranscripts is one of the key indicators of assembly quality and corresponds to the number of contigs that were either extended or had missing sequences recovered during assembly improvement. Completeness was evaluated using the number of bases present in the assembly, mean length of the contigs and the proportion of the reference supertranscriptome bases present in the assembly (reference coverage) calculated using TransRate (Table 3). Both mean contig length and reference coverage were higher in the improved assembly compared to the initial assembly for all species. Number of bases in the assembly was also found to be higher in all the improved assemblies except that for Arabidopsis. On investigating we found that this was due to the presence of high number of redundant contigs ($\sim$ 6900, 13.8%) in the Arabidopsis assembly, which were removed by CD-HIT-EST before the start of the iterative improvement. In other species, the number of redundant contig was between 0.01%-$-$0.06% (see Additional file 1: Table S1). Taken together these metrics suggest a better representation of supertranscripts in the assemblies resulting after improvement with ROAST.

**Table 3:** Evaluation of ROAST using real RNA-seq datasets

Organism	Assembly	Completeness			Fragmentation		False chimera	Inversion/translocation	Overall quality
		No. of bases $^\text{a}$	Mean contig length $^\text{b}$	Reference coverage $^\text{c}$	BLAST analysis	Proportion of read pairs on different contigs $^\text{b}$	Chimeric contigs $^\text{c}$	Proportion of read pairs with incorrect orientation $^{b*}$	TransRate score $^\text{a}$
Human	Initial	46,096,587	1109	0.09	3898	0.038	**2184**	**0.011**	0.46
	Improved	**49,024,824**	**1271**	**0.10**	**3709**	**0.029**	2529	0.013	**0.51**
Mouse	Initial	29,591,694	1131	0.07	2970	0.086	**851**	0.004	0.24
	Improved	**30,458,066**	**1216**	**0.08**	**2830**	**0.060**	877	**0.003**	**0.28**
Chicken	Initial	54,635,372	1018	0.21	**2330**	0.033	1747	0.003	0.51
	Improved	**57,983,502**	**1072**	**0.23**	2380	**0.019**	**1690**	**0.002**	**0.55**
Rice	Initial	26,514,527	701	0.17	2083	0.048	1253	0.002	0.40
	Improved	**30,336,262**	**784**	**0.18**	**1790**	**0.039**	**1151**	**0.001**	**0.44**
Arabidopsis	Initial	**32,430,389**	646	0.24	11,373	0.067	4697	0.011	0.16
	Improved	30,336,262	**675**	**0.27**	**10,030**	**0.055**	**3725**	0.011	**0.40**

Superior values shown in bold face Values calculated using:^a^ TransRate, ^b^ Samtools, ^c^ rnaQUAST.

* F1F2 and R1R2 orientations

Another parameter that provides key insights into assembly quality is the contiguity of supertranscripts. This corresponds to the number of fragmented contigs present in the assembly and is negatively correlated with the assembly quality. We calculated the number of fragmented contigs using two strategies. First, BLAST-based assessment method was used to search the assembled supertranscriptomes against the reference supertranscriptomes. Two contigs were regarded as fragmented if they showed 90% or more query coverage per subject against the same reference supertranscript and the length of query supertranscript covered at least 10% of the length of reference supertranscript. As can be seen from Table 3, the number of fragmented supertranscripts were reduced in all cases except chicken after improvement with ROAST. Increase in the fragmented supertranscripts count in chicken can be explained by the fact that extension of partial supertranscripts during assembly improvement resulted in 659 additional contigs falling in the criteria of 90% or more query coverage per subject compared to the initial incomplete contigs leading to the identification of more fragmented supertranscripts in the improved assembly (see Additional file 1: Table S2). Another metric for assessing the contiguity of supertranscripts is the proportion of read pairs mapped on different contigs (see the paragraph “Reads with mates mapped on a different contig” above). An assembly containing a higher number of fragmented supertranscripts will have greater fraction of reads with mates mapped on a different contig compared to the one with a lower fragmented supertranscripts count. The proportion of read pairs mapped on different contigs showed remarkable decrease for improved assemblies in all species compared to the initial assemblies (Table 3) thus implying a reduction in the number of fragmented supertranscripts in the assemblies post ROAST optimization.

To assess the decrease in false chimeras and local mis-assemblies including inversions and translocations, we used the number of chimeric contigs reported by rnaQUAST and the proportion of read pairs mapped in incorrect orientation (F1F2 and R1R2), calculated using Samtools respectively (Table 3). These parameters also negatively correlate with the assembly quality. The number of chimeric contigs were found to be reduced in three out of five species including chicken, rice and Arabidopsis. Creation of new chimeric contigs (for example, in the case of human and mouse) due to erroneous merging of fragmented supertranscripts can be avoided by increasing the length of soft-clipped bases mapping on different contigs, number of reads containing soft-clipped bases, number of reads with mates mapped on different contigs and/or setting a higher BLAST threshold to find overlap between two identified contigs as fragments. However, it must be kept in mind that a more stringent criteria for these parameters may lead to fewer merging of original fragmented supertranscripts. Inversions and translocations were also found to be reduced in the ROAST improved assemblies in mouse, chicken and rice while in Arabidopsis no change in the proportion of incorrectly mapped read pairs was observed. In human, on the other hand, slightly higher proportion of read pairs mapped in incorrect orientation was observed suggesting further occurrence of false inversions and/or translocations. Like chimeric contigs, this can be improved by setting a higher soft-clipped base support for the position, soft-clipped consensus sequence length threshold and BLAST score to identify and fix local mis-assemblies and merge overlapped edges of fragmented supertranscripts. It must, however, be noted that using too stringent criteria may lead to the missing of true assembly errors by ROAST.

**Fig. 4:**
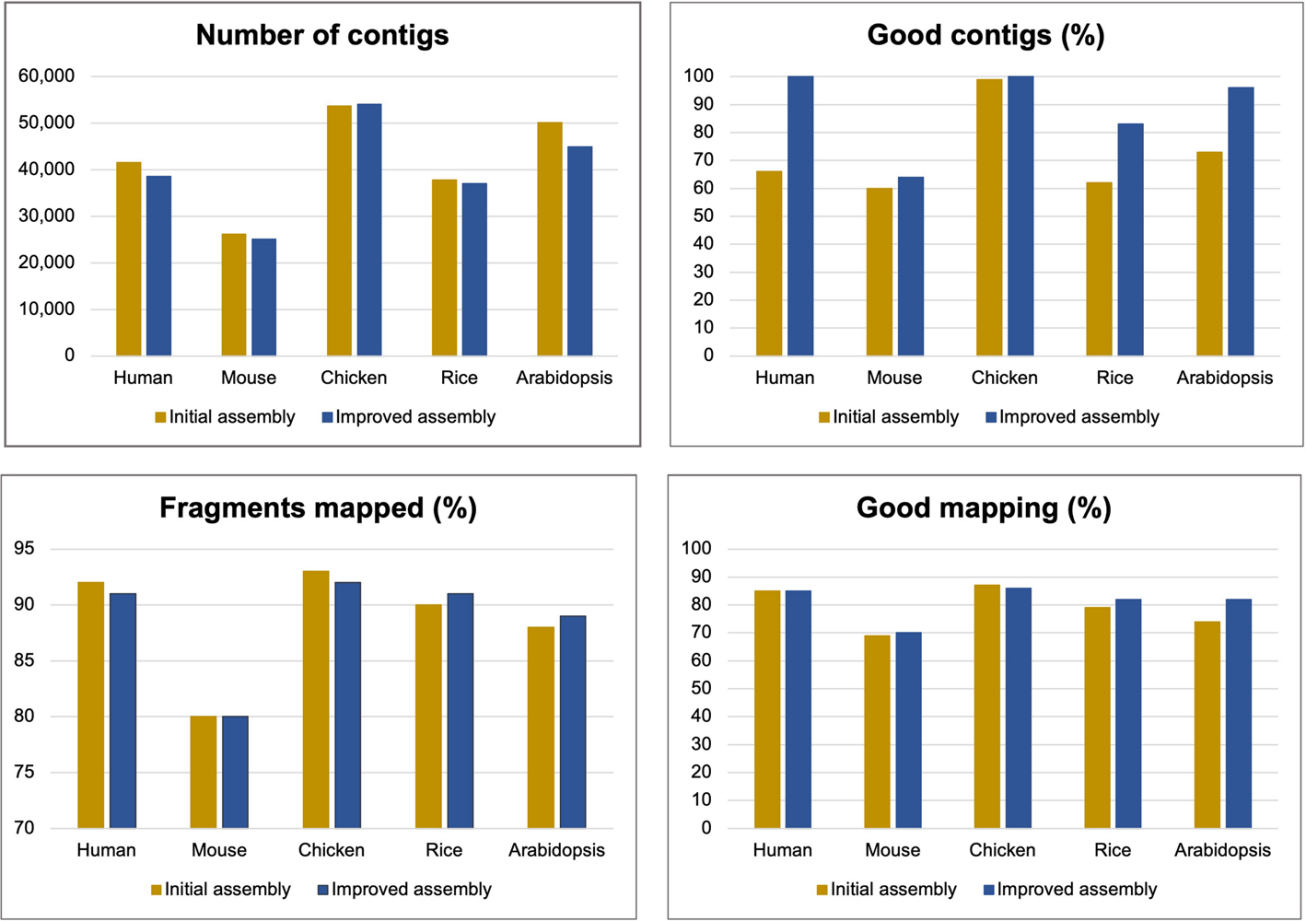
Assessment of assembly quality before and after improvement using ROAST. Initial and ROAST-optimized assemblies were compared using different metrics reflecting overall assembly quality. These include number of contigs present in the assembly, percentage of reads mapped, and percentage of contigs and aligned reads regarded as ‘good’ by TransRate [22]. See text for details on how contigs and reads are classified as ‘good’ by TransRate

Finally, the overall assembly quality was evaluated using the TransRate score (see the section “Evaluation using simulated datasets” above) as well as different assembly-level metrics. In all cases, TransRate score was higher after ROAST optimization (Table 3) indicating that ROAST was able to improve overall assembly quality by identifying and fixing various types of assembly errors. Besides TransRate score, there are various metrics that can be used to assess overall assembly quality. These include number of contigs present in the assembly reflecting its compactness, percentage of reads mapped, and percentage of contigs and aligned reads regarded as ‘good’ by TransRate. ‘Good contigs’ are determined by TransRate using a cutoff optimization procedure for individual scores of the contigs calculated using factors described above while ‘Good mapping’ correspond to those alignments that are consistent with a perfectly assembled contig, for example read pairs mapped in correct orientation on the same contig without any anomaly [22]. These metrics are shown in Fig. 4 for initial and improved assemblies. The number of contigs were lower while the percentage of good contigs increased in the improved assembles. Similarly, the percentages of reads mapped and good mapping also went up after ROAST optimization. Overall, these results suggest that ROAST is able to produce assemblies which have fewer redundancies and a better representation of supertranscripts compared to the their initial versions.

As ROAST relies solely on RNA-seq data for assembly improvement, its efficacy in improving the quality of supertranscripts depends on the read abundance for that supertranscript. Highly abundant supertranscripts are subject to more error fixation and improvement in quality compared to the ones with same number of errors but have low read abundance. Similarly, an assembly with lower read count might have fewer errors fixed compared to the one with higher number of reads since many error signatures do not meet the default cut-offs. To circumvent this, ROAST allows users to adjust different parameters such as setting lower threshold for reads with partially mapped mates at the edges of the contig and reads with unmapped mates to identify and extend incomplete supertranscripts. Similarly, setting lower thresholds for reads partially mapped with soft-clipped bases mapping on different contigs, reads with mates mapped on a different contig and reads partially mapped inside a contig can help in identifying and merging fragmented supertranscripts, and fixing local mis-assemblies and false chimeras when read coverage is low in the RNA-seq data. However, it must be kept in mind that using very low threshold values may result in the over-correction of assemblies. Also, many of these parameters are interrelated and changing one parameter to reduce a particular error type may also result in the reduction of number of errors being fixed for other types, or may lead to an overly corrected assembly containing additional assembly errors as discussed above. Moreover, since each dataset differs in complexity, it might be useful to evaluate different parameter combinations to identify what works best for the supertranscriptome assembly being optimized. Under default settings ROAST runs until no more assembly error can be identified or a maximum of 100 iterations is reached. For the actual datasets, it took ROAST between 19 (Arabidopsis) and 39 (mouse) iterations for assembly improvement (see Additional file 1: Table S3). The number of iterations taken depends on the complexity as well as extent of abnormalities in the assemblies. This default behaviour can be changed by increasing the threshold for leftover errors or reducing the maximum number of allowed iterations at the cost of final assembly quality.

## Conclusion

Here, we present ROAST a tool to identify and fix supertranscriptome assembly errors including missing sequences and various structural anomalies including fragmented supertranscripts, false chimera, inversions and translocations produced by current assemblers using Illumina paired-end sequencing data without the aid of reference sequence. Since ROAST does not rely on running BLAST using closely related species to improve the reference supertranscriptome, it is highly useful for studies involving non-model organisms where a high quality reference genome or transcriptome may not be available for closely related organisms. ROAST identifies and fixes the assembly errors using the paired-end information of the reads and the error signatures produced during read alignment including soft-clipped bases, unexpected change in expression coverage, and reads with mates unmapped or mapped on a different contig. At the start of each iteration, improved assembly from the previous iteration serves as a reference for the current iteration for aligning RNA-seq reads and identifying error signatures thereby allowing maximum improvement in the reference sequence. In addition to its core algorithm for error identification and correction, ROAST uses a number of tools during the iterative improvement process. All these tools contribute the overall running time of ROAST as well as the extent of assembly improvement made by ROAST. Test runs on both simulated and real datasets show that ROAST significantly improves assembly quality by iteratively reducing assembly errors from the reference sequence. Supertranscriptome assemblies resulting ROAST processing provide a better representation of the underlying transcripts than those without any refinement, and are useful in a wide range of studies including homology inference for phylogenetic analysis, metabolic pathway reconstructions, metabolic flux analysis and differential expression analysis [18]. Hence, ROAST can be used as a downstream improvement step of *de novo* supertranscriptome assembly algorithms to help improve the quality of assembled supertranscriptome by fixing common assembly errors.

## Methods

### Construction of reference supertranscriptomes

Reference supertranscriptomes were constructed for human (*Homo sapien*), mouse (*Mus musculus*), chicken (*Gallus gallus*), rice (*Oryza sativa*) and Arabidopsis (*Arabidopsis thaliana*) using their reference genomes. Reference genomes were obtained from Ensembl (https://www.ensembl.org) for human (GRCh38), mouse (GRCm38), chicken (GRCg6a), rice (IRGSP$-$1.0) and from TAIR (https://www.arabidopsis.org) for Arabisdopsis (TAIR10). The transcriptomes were converted into supertranscriptomes using the script provided by Davidson et al., 2017 [8], which creates a reference supertranscript for each gene by concatenating the exonic sequences for the gene.

### Simulated RNA-seq data

Simulated RNA-seq reads for all model organisms used in this study were generated from their respective reference supertranscriptomes using Mason2 [39]. A total of 80 million reads (40 million read-pairs) of length 100bp were simulated with options “-illumina-prob-mismatch-scale 2.5 -fragment-max-size 500 -fragment-min-size 250”. The number of reads was chosen to keep the size of the simulated datasets close to that of actual datasets, which is  40 million read pairs.

### Simulated assembly errors

A total of 800 simulated errors (200 errors for each error type) were produced in each reference supertranscriptome. Partial supertranscripts were generated by removing 10 to 30 percent of a contig from one or both sides of the supertranscript. To produce fragmented supertranscripts, a supertranscript was broken into two at a randomly sampled position within 40% and 60% of the contig. Similarly, false chimeras were generated by breaking and fusing two randomly selected contigs. Local mis-assemblies were produced as follows. A randomly selected fragment of length between 30% and 70% of read length was removed from or added to a supertranscript to mimic a missing sequence or an unsupported insertion. Finally, translocations and inversions were simulated by removing a fragment of length equal to 20–30% of supertranscript length from a supertranscript and added at a different position within the same supertranscript in the same orientation (for translocation) or reverse orientation (for inversion). A total of 50 errors were produced for each type of local mis-assembly. In all cases, contigs were randomly selected as long as they were $>500$ bp in length and contained no low quality alignment (Phred score $< 20$). To avoid a situation whereby contigs containing simulated errors were marked as redundant by CD-HIT-EST and, consequently, removed at the beginning of the assembly improvement (see the section “ROAST overview” Section above), supertranscripts having $\ge 95\%$ similarity were ignored when producing an error and so were the overlapping genes.

### Real datasets

To demonstrate the utility of ROAST for obtaining an improved reference assembly for benchmark data, real datasets for human, mouse, chicken, rice and Arabidopsis were used. These datasets have been previously used in benchmark comparisons in different studies [22, 23, 31]. The datasets were obtained from NCBI Sequence Read Archive database using accession numbers SRR493369-SRR493371 (human), SRR203276 (mouse), SRR1956755 (chicken), SRR037735-SRR037738 (rice) and SRR1655112 (Arabidopsis), and consisted of paired-end reads of length 75–101 bp generated using Illumina paired-end sequencing technology.

### De novo supertranscriptome assembly of model organisms

De novo supertranscriptome assemblies of the model organisms were generated using the benchmark datasets using Trinity v2.11 [3] with default parameters and the flag “–include_supertranscripts”. The supertranscriptome assemblies were used as input along with respective paired-end RNA-seq data to identify and fix assembly errors and improve assembly quality using ROAST.

### Alignment, filtering and processing of RNA-seq data

During the iterative improvement process, ROAST aligns the RNA-seq data using Minimap2 v2.17 [40], which allows soft-clipped bases during mapping, and HISAT2 v2.0.4 [41], a splice-aware aligner, to get splice site information. Read with low mapping quality (Phred score $< 20$) and read pairs mapped in incorrect orientations including F1F2, R1R2, and R1F2 are removed before further processing. Base coverage data, required for error identification and fixation, is generated using Samtools v1.9 [38] from the filtered alignment files. Picard tool (https://broadinstitute.github.io/picard/) is used to generate FASTQ files using the reads mapped at contig edges during each inner iteration (see the Paragraph “Reads partially mapped at the edges of a contig” above). To distinguish chimeric positions from exon-exon split boundaries, ROAST uses the Cufflinks v2.2.1 [42] on the alignment file generated by HISAT2. CAP3 assembler [30] is used to generate consensus sequence from partially mapped reads and to construct assemblies from unmapped read for the extension of partial supertranscripts.

### Removal of redundant contigs

At the start of the iterative improvement process, ROAST removes redundancies between contigs using CD-HIT-EST v4.8.1 [26] with sequence identity cut-off 0.95, a commonly used threshold [12, 43–45].

### Software availability

ROAST is written in C++ and uses two external libraries. These include Bamtools, a C++ API and toolkit to analyze and manage BAM files [46], and Boost [47]. It also uses a number of tools including CD-HIT-EST, Minimap2, HISAT2, Cufflinks, BLAST (blastn), Picard tool and CAP3 for read alignment, filtering and local assembly. The source code, released under open source MIT license, and pre-compliled binaries of external tools required by ROAST are available for download at https://github.com/azizmithani/roast/. The tool can be run via a command line interface on Linux machines.

### Supplementary Information

**Additional file 1**. Supplementary Figures S1 to S17 and Supplementary Tables S1 to S3.

## Data Availability

The test dataset used during the current study and results obtained are available in Zenodo repository (https://zenodo.org/record/8192067). ROAST is released under open source MIT license and is available at https://github.com/azizmithani/roast. Project name: ROAST. Project home page: https://github.com/azizmithani/roast. Operating system(s): Linux. Programming language: C++. Other requirements: Java, Python, C++, BLAST, Samtools (version >= 1.9), BOOST API library, Bamtools API library. License: Open source MIT license. Any restrictions to use by non-academics: None. Publicly available datasets were used in this study to assess performance of ROAST. The datasets were obtained from NCBI Sequence Read Archive database using accession numbers SRR493369-SRR493371 (human), SRR203276 (mouse), SRR1956755 (chicken), SRR037735-SRR037738 (rice) and SRR1655112 (Arabidopsis).

## Abbreviations

ROAST: Reference-free optimization of assembled supertranscriptomes
STs: Supertranscripts
NGS: Next generation sequencing
IUPAC: International union of pure and applied chemistry
