# Supplementary material for: Roast: a tool for reference-free optimization of supertranscriptome assemblies
Source: BMC Bioinformatics. 2024 Jan 2;25:2. doi: 10.1186/s12859-023-05614-4 (PMC10763045; doi:10.1186/s12859-023-05614-4)
Supplement: Supplementary file 1 — Additional file 1. Supplementary Figures S1 to S17 and Supplementary Tables S1 to S3. [file 12859_2023_5614_MOESM1_ESM.pdf]

Supplementary Figures and Tables for:

ROAST: a tool for reference-free optimization of supertranscriptome assemblies

Madiha Shabbir and Aziz Mithani

Department of Biology, Syed Babar Ali School of Science and Engineering, Lahore University of Management Sciences (LUMS), DHA 54792, Lahore Pakistan

Corresponding author: aziz@cantab.net

Contents:

ROAST Algorithm 2

- Supplementary Figure 1
- Supplementary Figure 2
- Supplementary Figure 3
- Supplementary Figure 4
- Supplementary Figure 5
- Supplementary Figure 6
- Supplementary Figure 7
- Supplementary Figure 8
- Supplementary Figure 9
- Supplementary Figure 10
- Supplementary Figure 11
- Supplementary Figure 12
- Supplementary Figure 13
- Supplementary Figure 14
- Supplementary Figure 15
- Supplementary Figure 16
- Supplementary Figure 17

Evaluation of ROAST using real dataset 19

- Supplementary Table 1
- Supplementary Table 2
- Supplementary Table 3

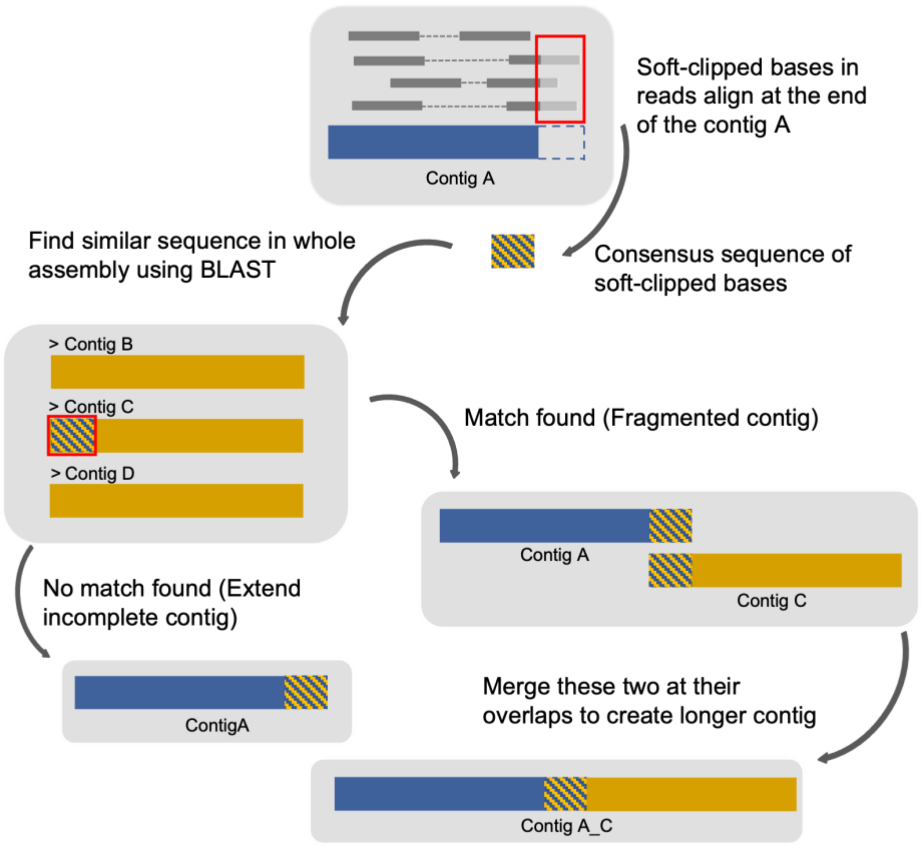

Supplementary Figure 1: Illustration of the algorithm for identification and fixation of incomplete and fragmented transcripts using soft-clipped bases.

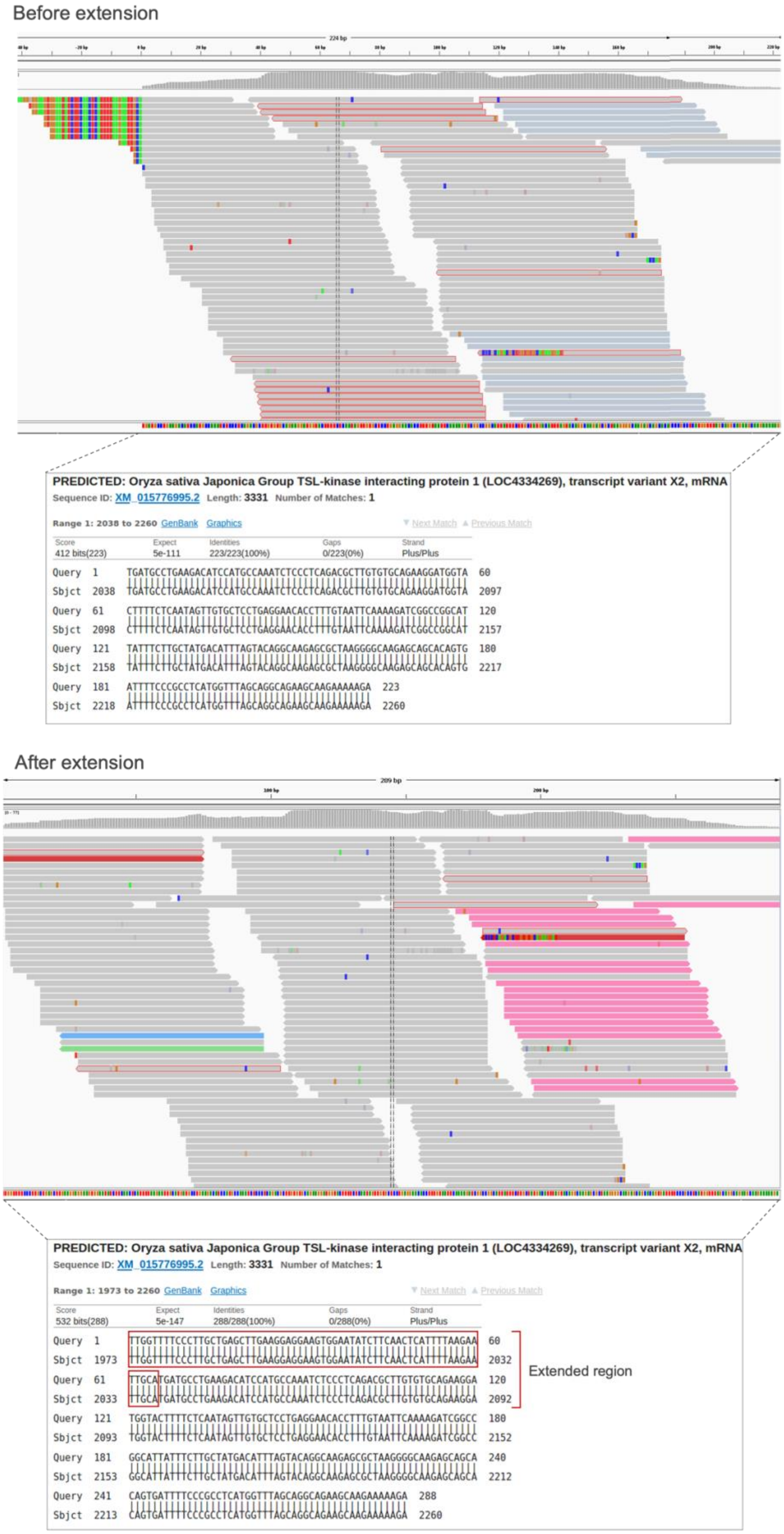

Supplementary Figure 2: An example showing identification and fixation of incomplete transcripts using soft-clipped bases.

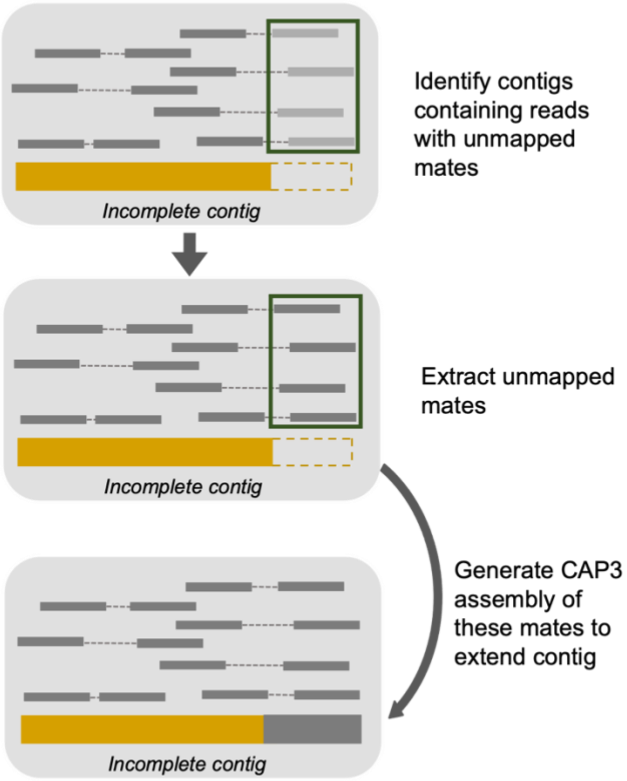

Supplementary Figure 3: Illustration of the algorithm for identification and fixation of incomplete transcripts using reads with unmapped mates.

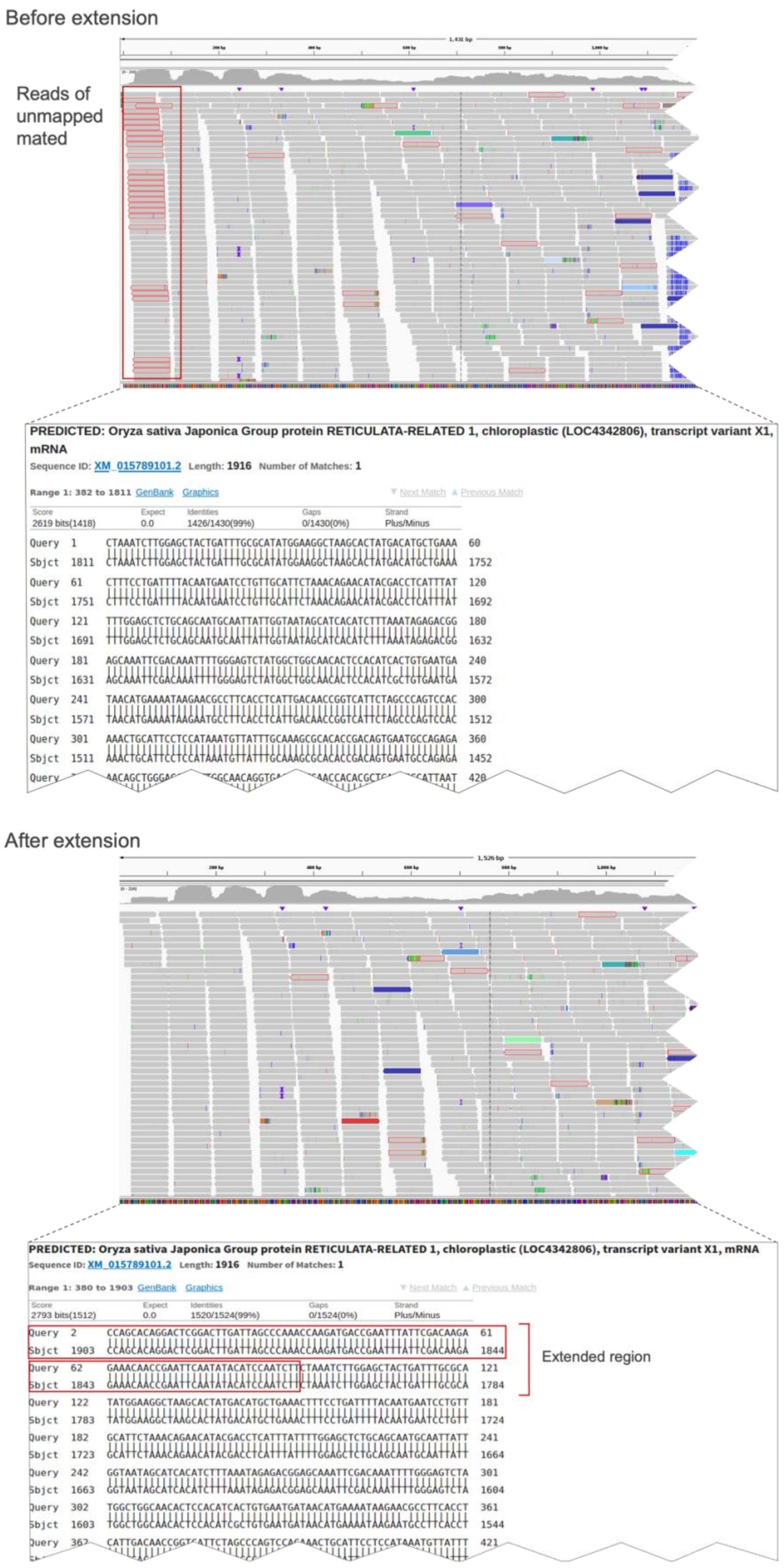

Supplementary Figure 4: An example showing identification and fixation of incomplete transcripts using reads with unmapped mates.

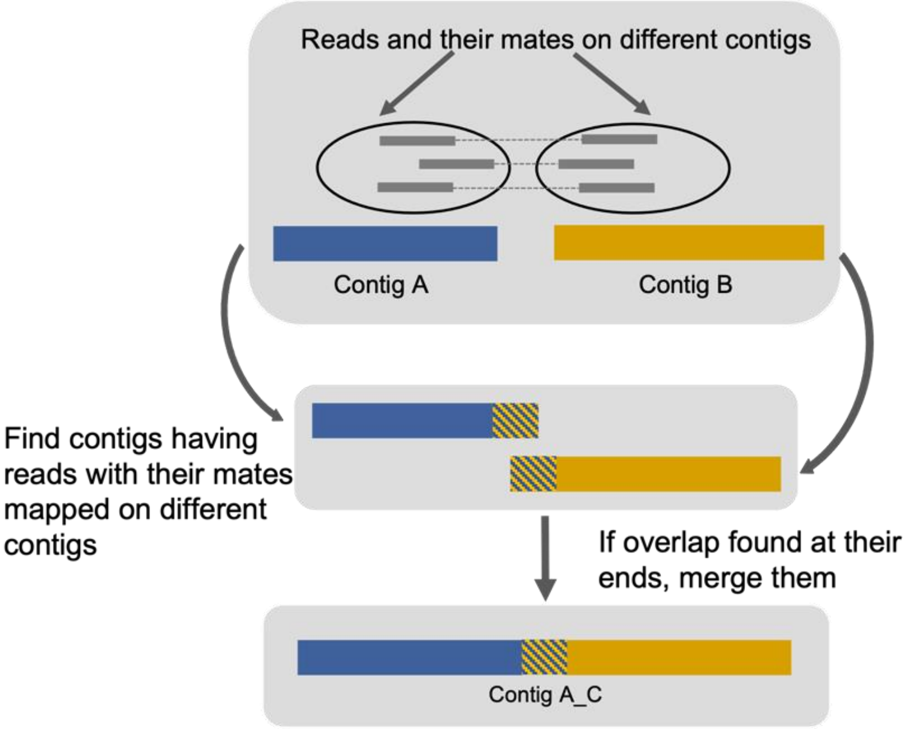

Supplementary Figure 5: Illustration of the algorithm for identification and fixation of fragmented transcripts using reads with mates mapped on different contigs.

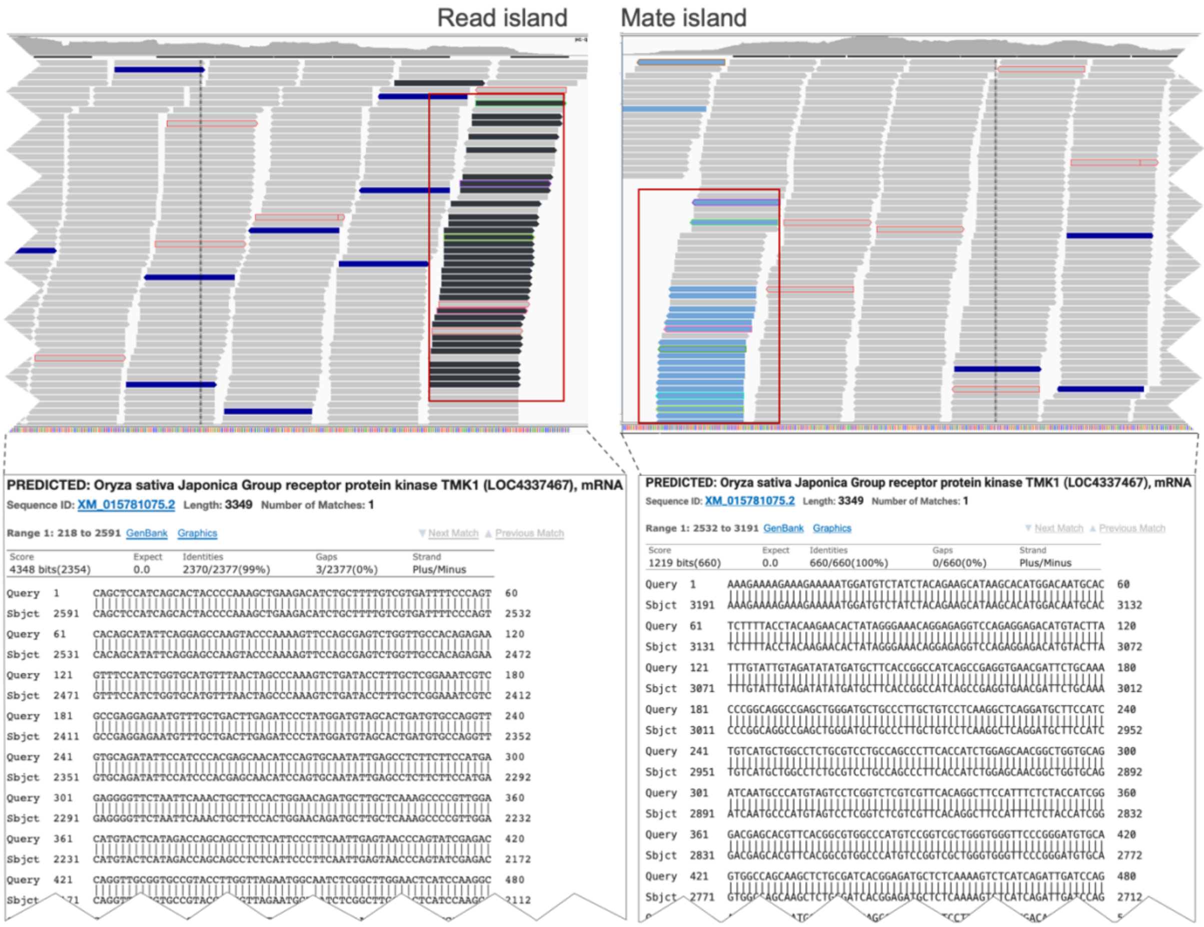

Supplementary Figure 6: An example showing identification and fixation of fragmented transcripts reads with mates mapped on different contigs.

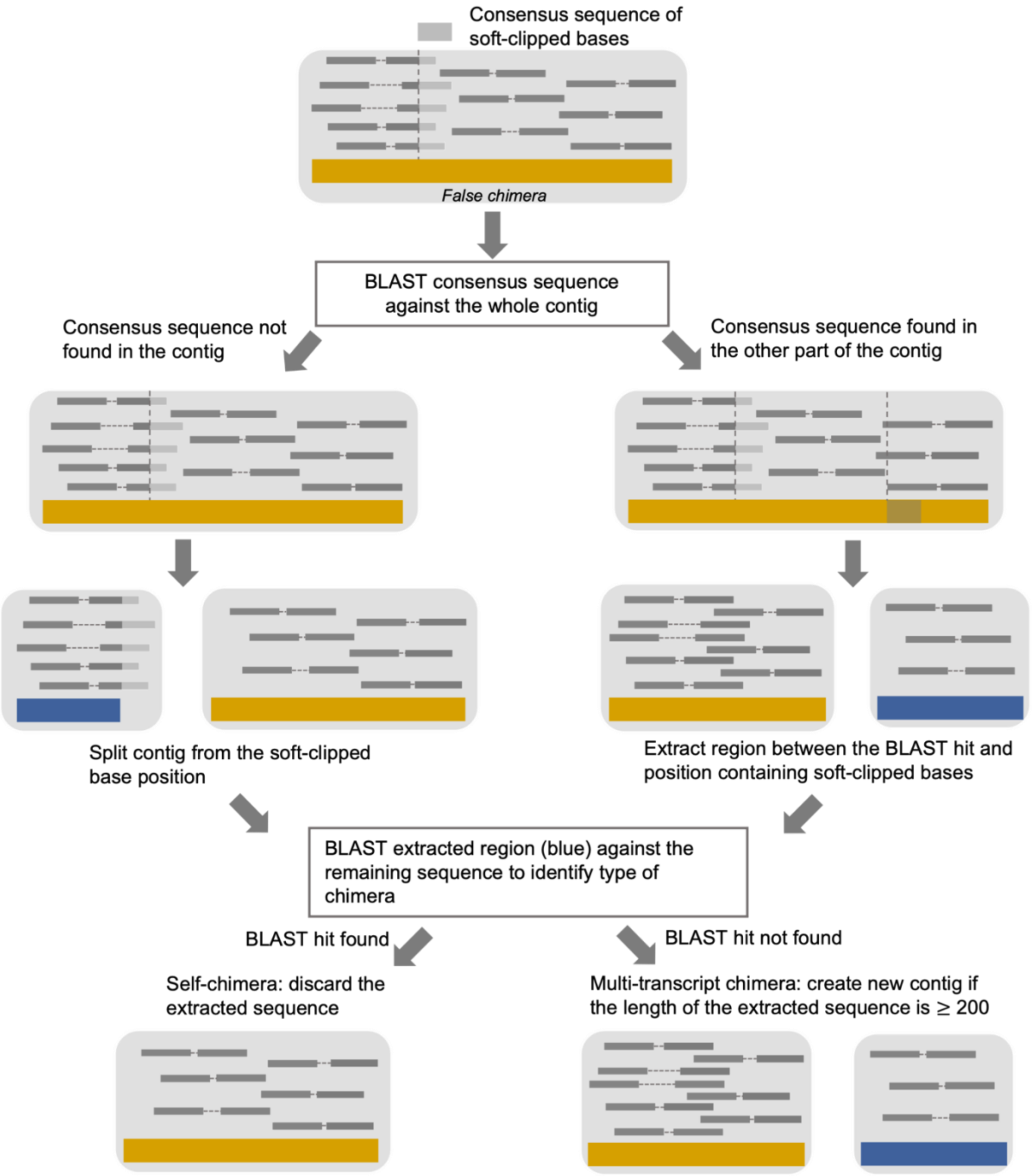

Supplementary Figure 7: Illustration of the algorithm for identification and fixation of false chimeras using reads partially mapped inside a contig.

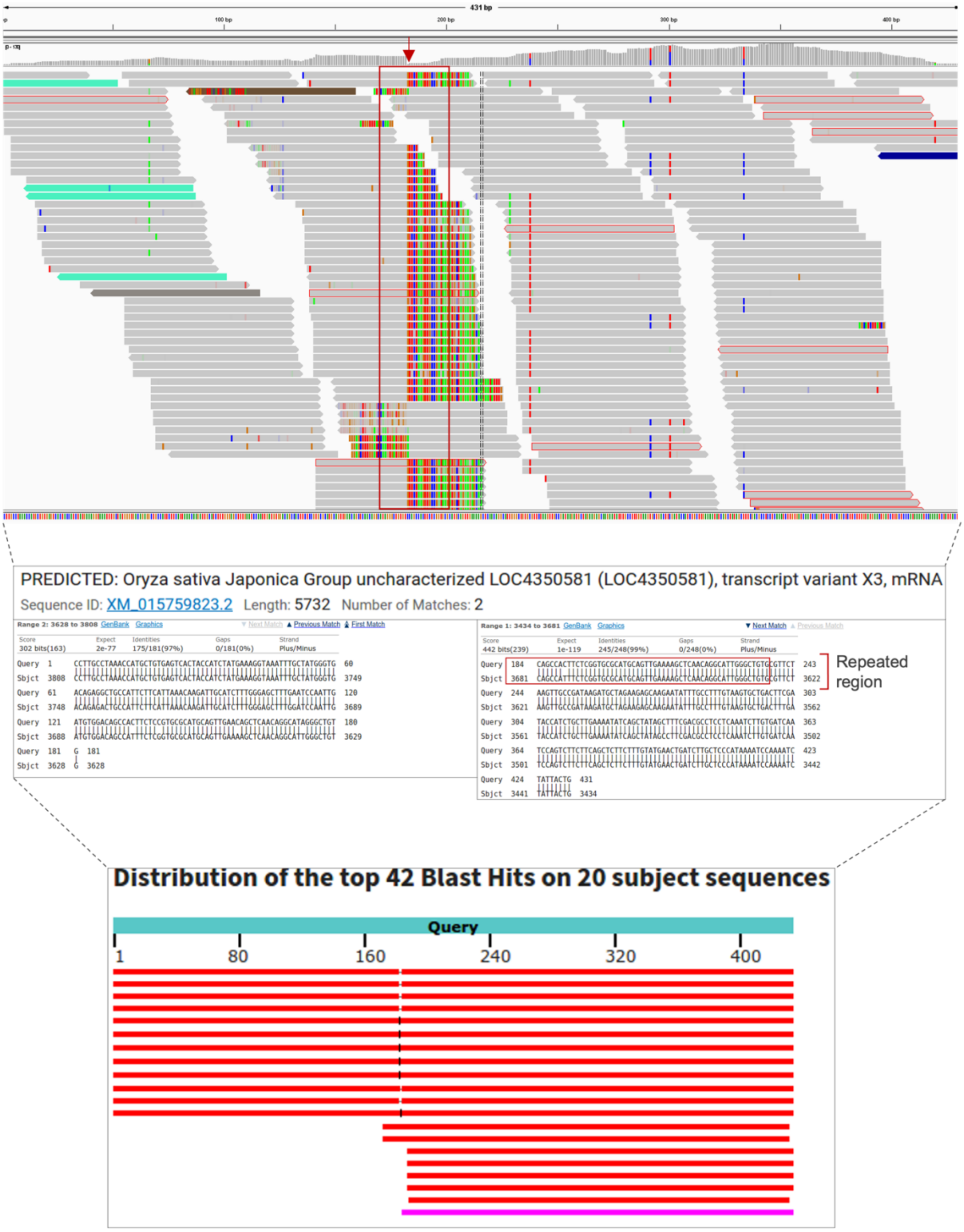

Supplementary Figure 8: An example showing identification and fixation of false self chimeras using reads partially mapped inside a contig.

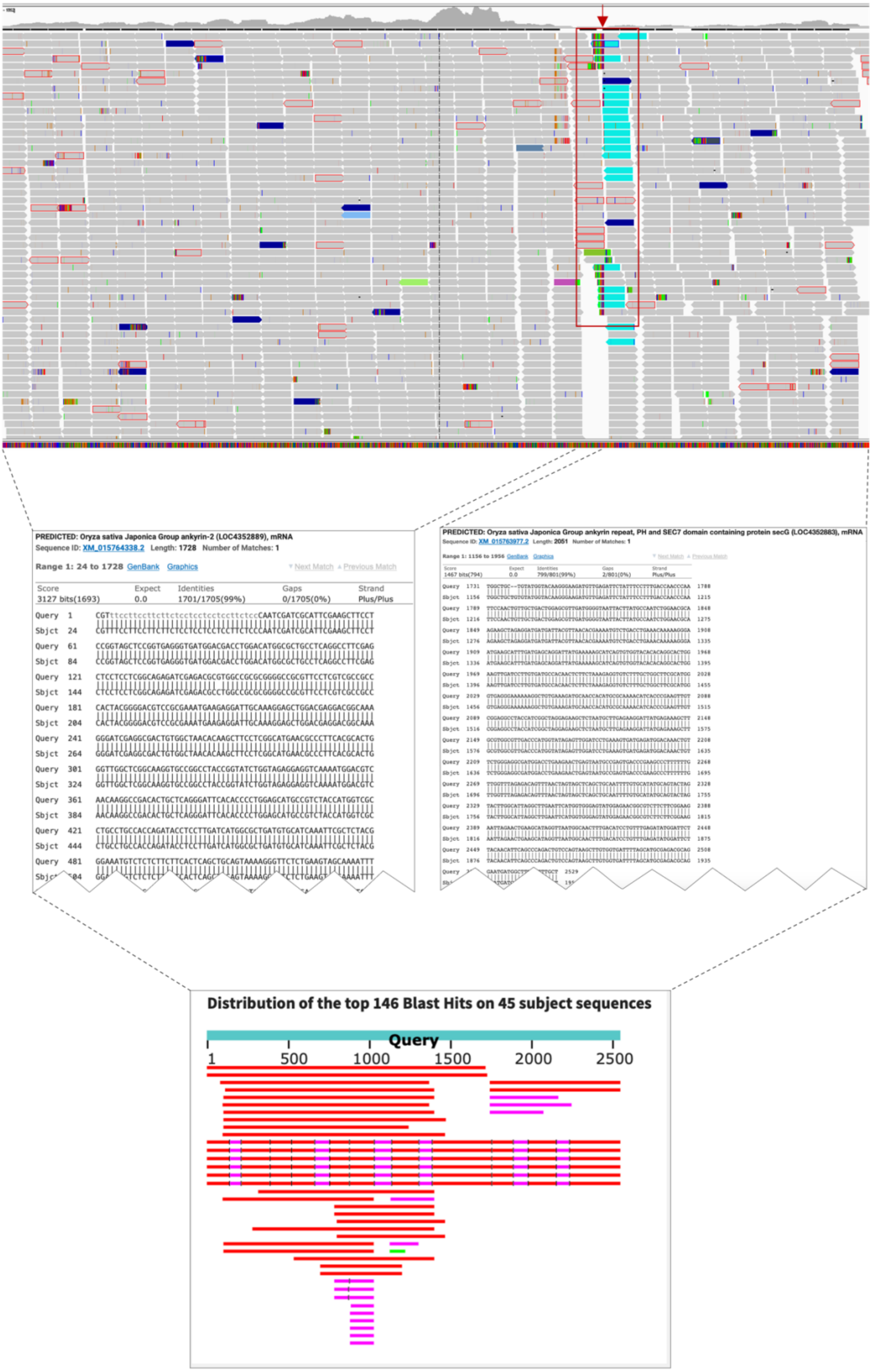

Supplementary Figure 9: An example showing identification and fixation of false multi-gene chimeras using reads partially mapped inside a contig.

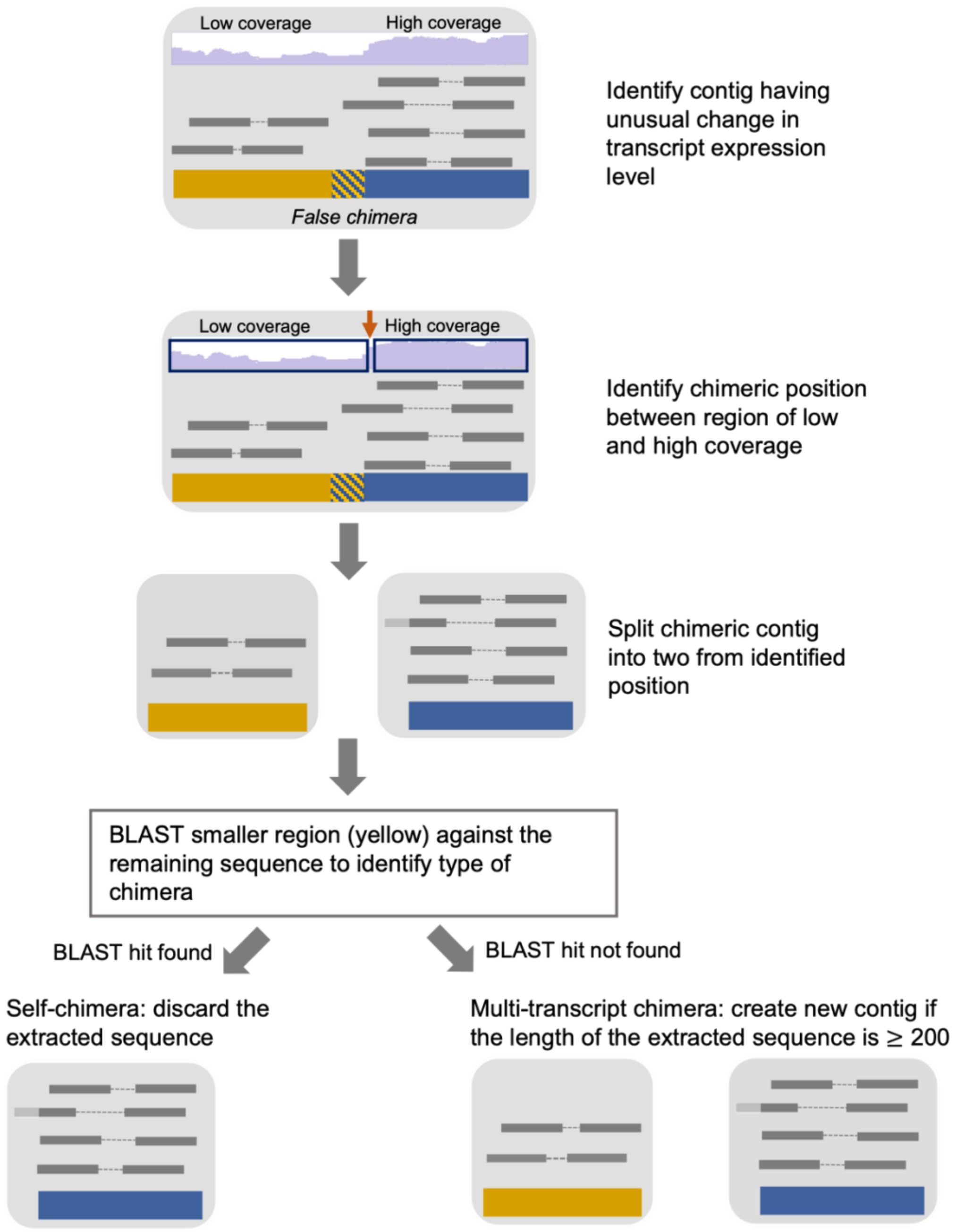

Supplementary Figure 10: Illustration of the algorithm for identification and fixation of false chimeras using unusual changes in the transcript expression levels.

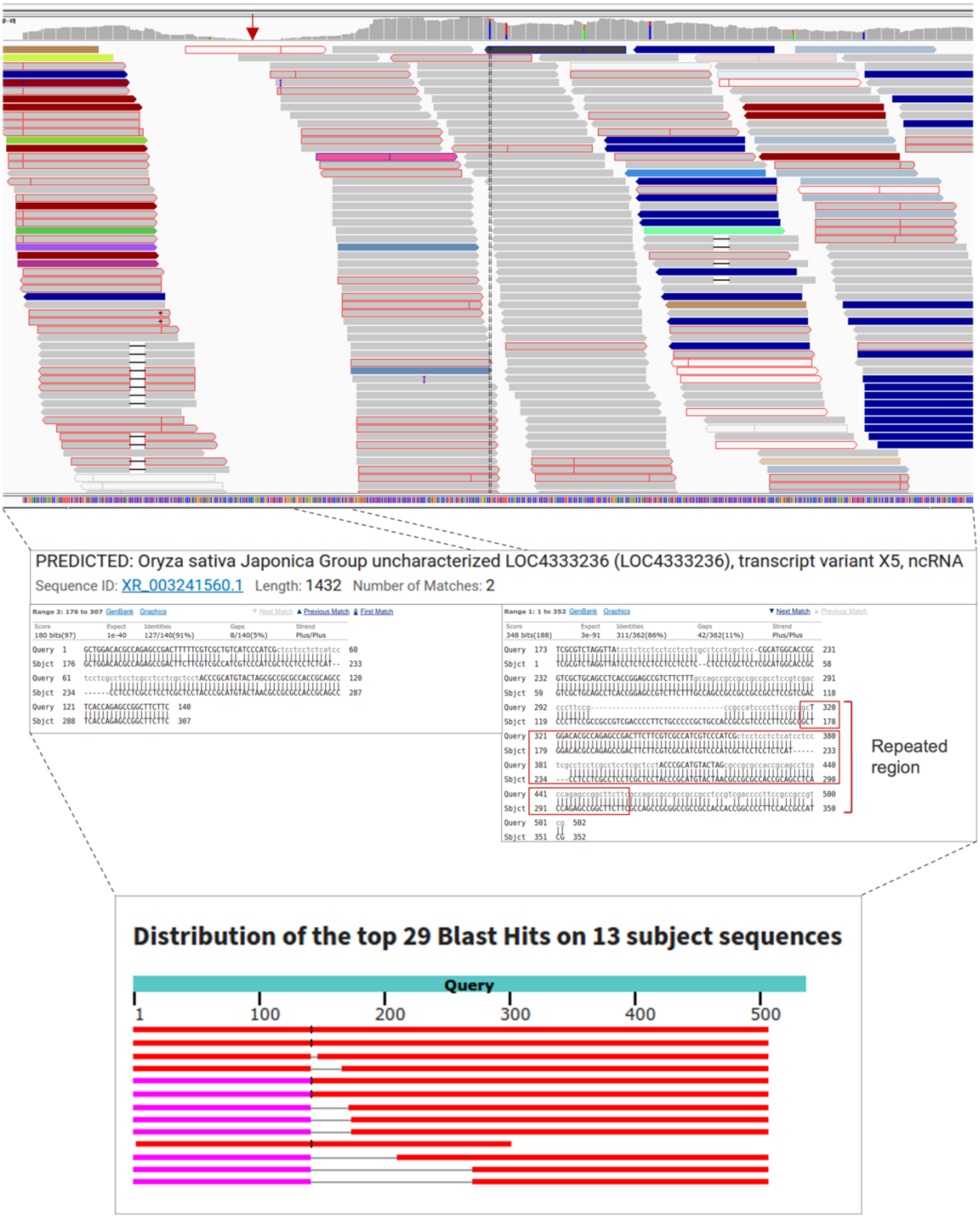

Supplementary Figure 11: An example showing identification and fixation of false self chimeras using unusual changes in the transcript expression levels.

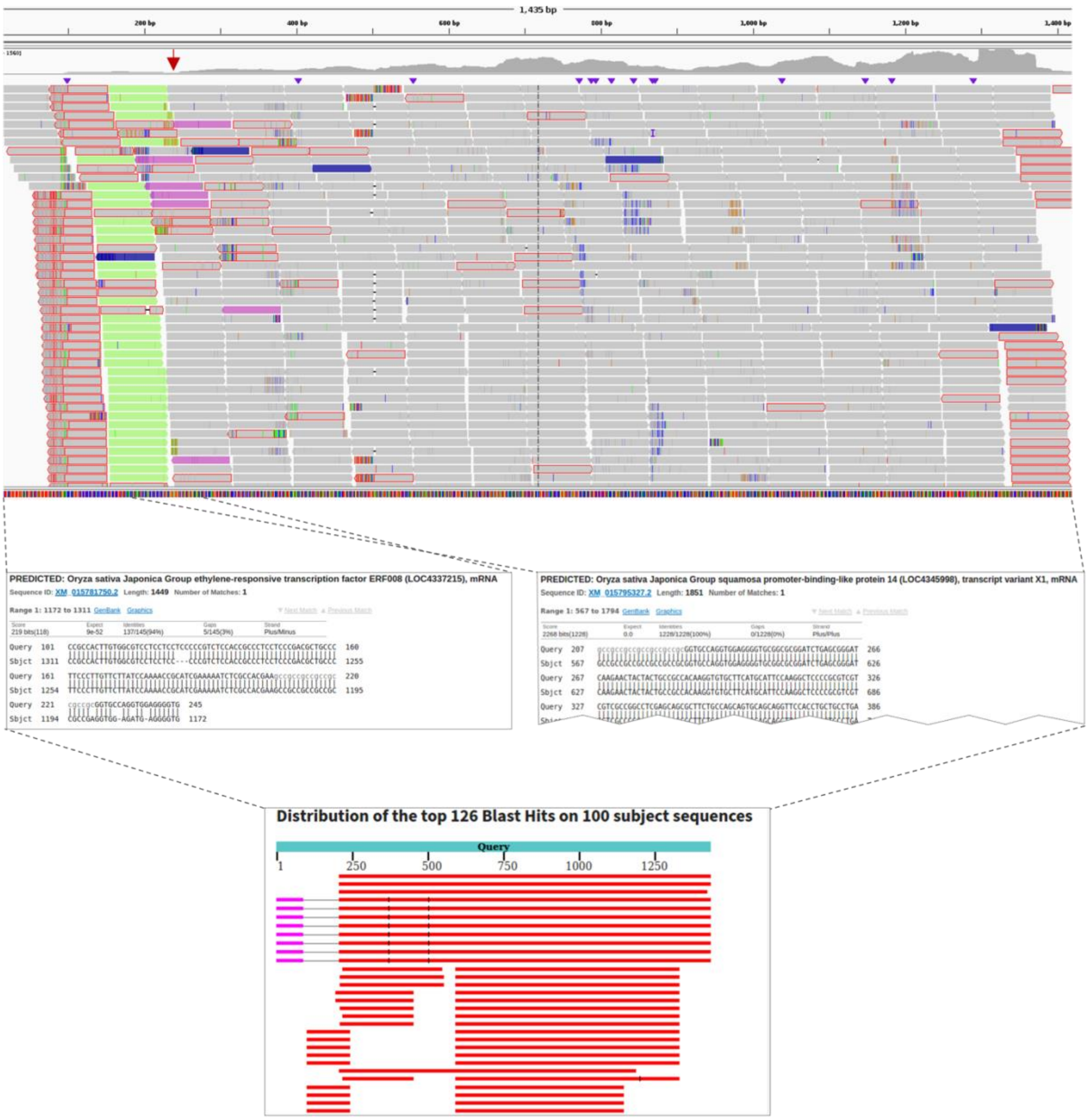

Supplementary Figure 12: An example showing identification and fixation of false multi-gene chimeras using unusual changes in the transcript expression levels.

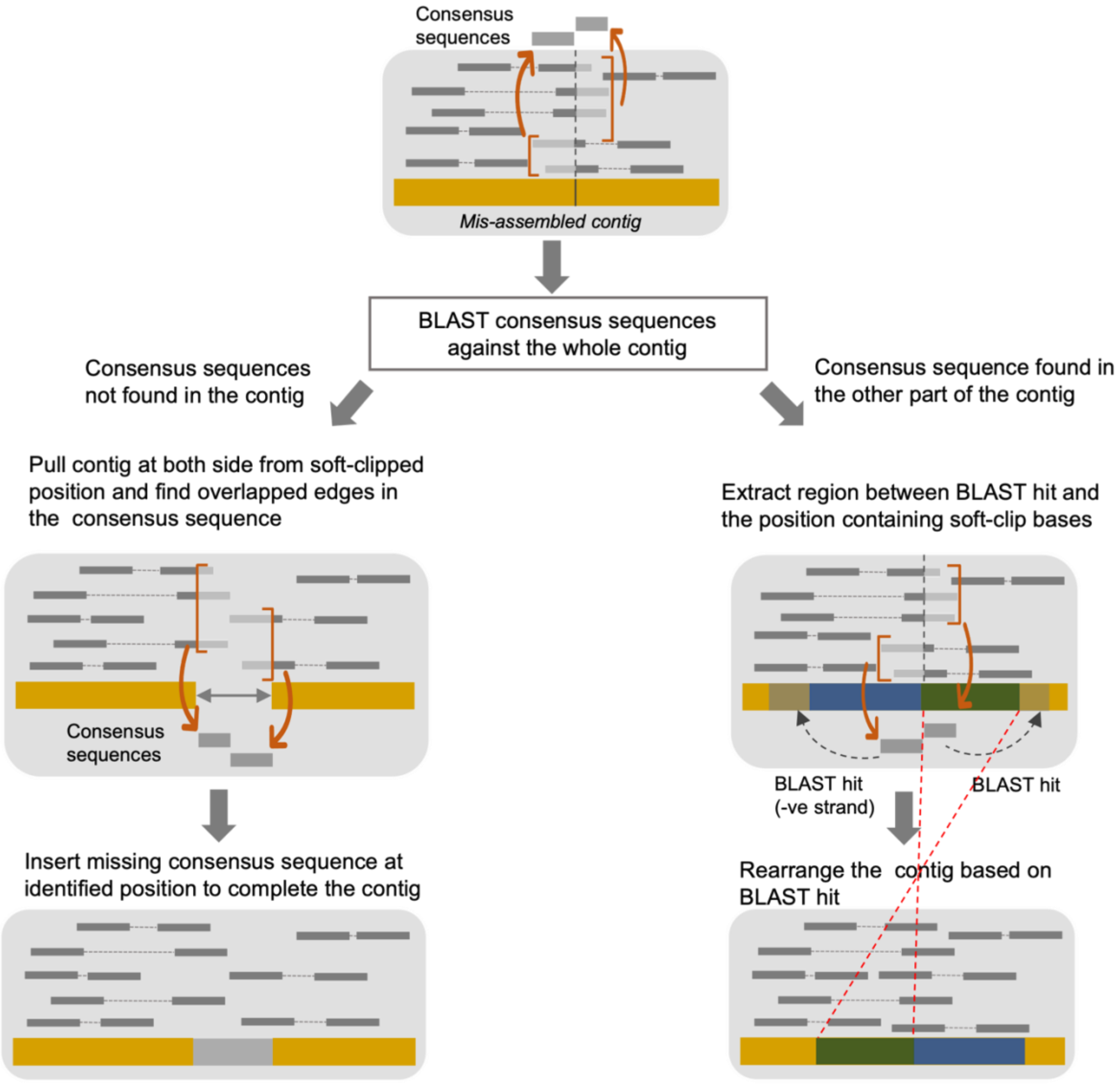

Supplementary Figure 13: Illustration of the algorithm for identification and fixation of missing sequences, translocations and inversions using soft-clipped bases facing in the opposite direction.

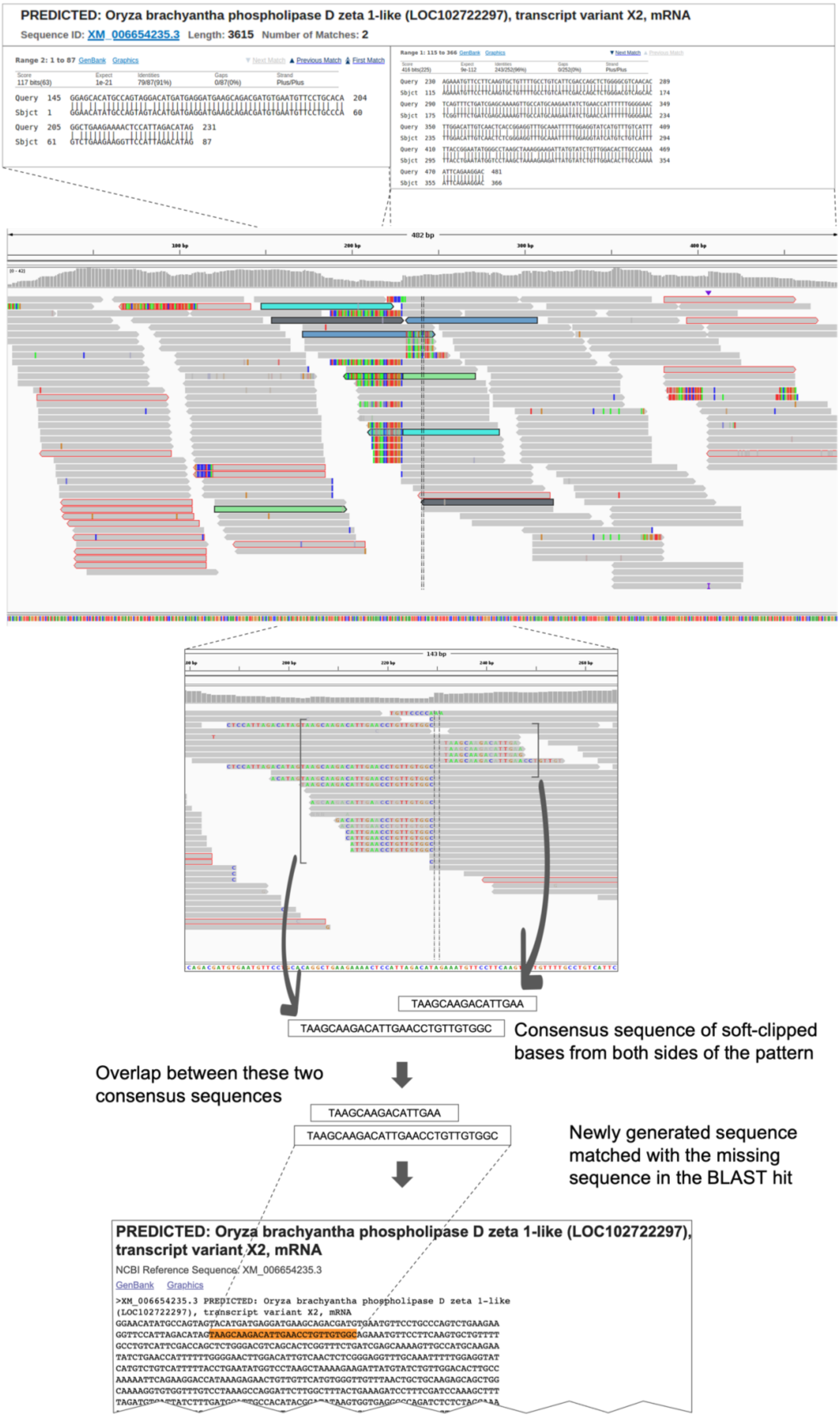

Supplementary Figure 14: An example showing identification and fixation of missing sequences using soft-clipped bases facing in the opposite direction.

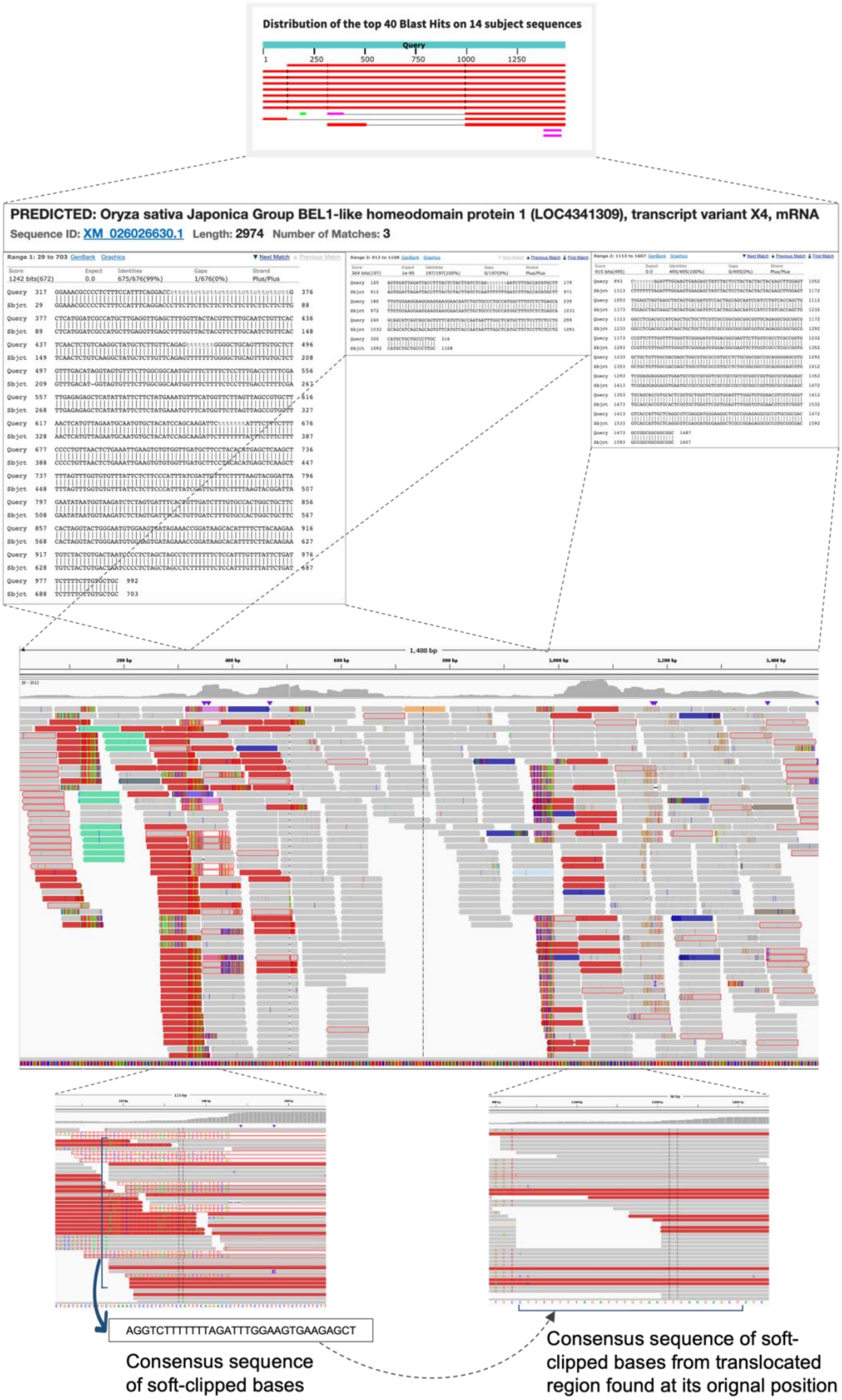

Supplementary Figure 15: An example showing identification and fixation of local translocation using soft-clipped bases facing in the opposite direction.

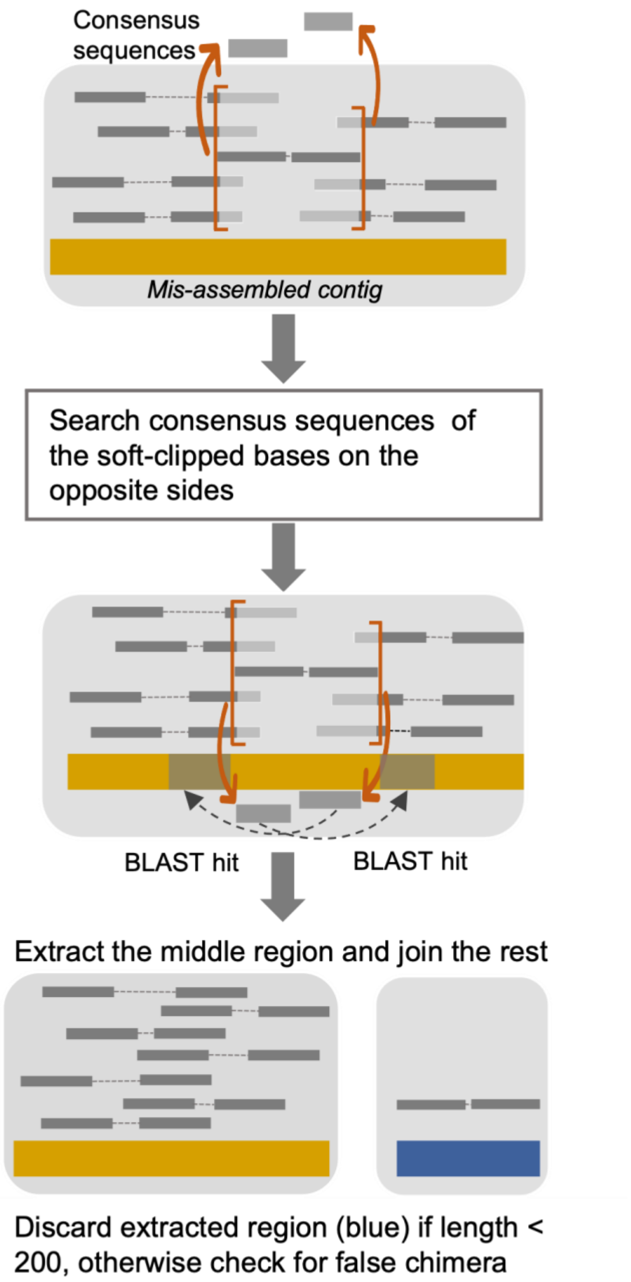

Supplementary Figure 16: Illustration of the algorithm for identification and fixation of unsupported insertions using soft-clipped bases facing each other.

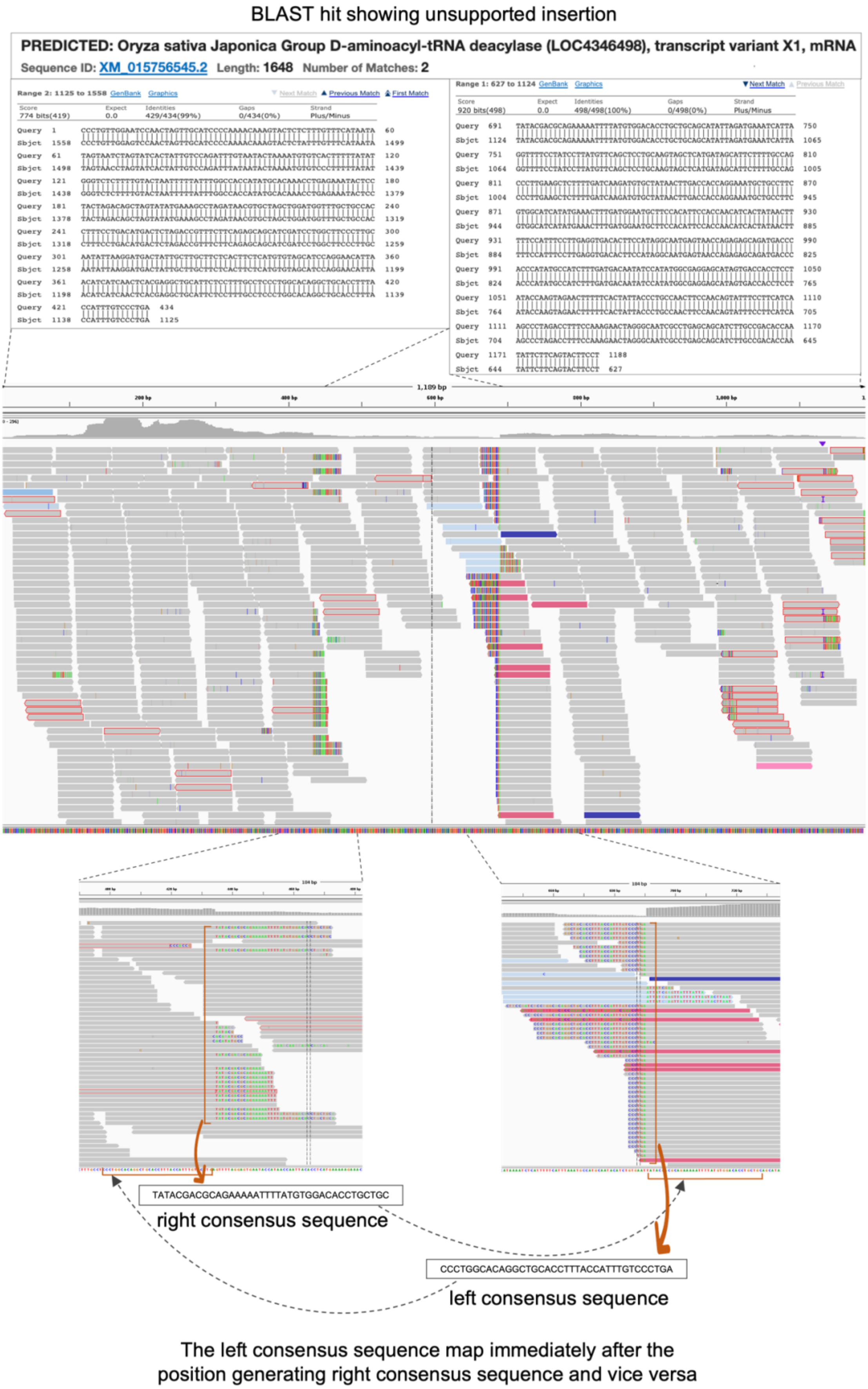

Supplementary Figure 17: An example showing identification and fixation of unsupported insertions using soft-clipped bases facing each other.

Table S1: No. of contigs removed from initial raw assemblies using CD-HIT-EST processing with ROAST

Species	# of contigs removed by CD-HIT-EST
Human	27(0.06)
Mouse	4 (0.01)
Chicken	9(0.016)
Rice	4 (0.01)
Arabidopsis	6916 (13)

Percentages are shown in paranthesis

Table S2: No. of contigs covered by refernce supertranscriptomes by 90% of their length before and after improving with ROAST

Species	Assembly	No. of contigs	Contigs covered by reference
Human	Initial	41,563	15,409 (37)
	Improved	38,586	15,366 (40)
Mouse	Initial	26,169	12,496 (47)
	Improved	25,055	12,388 (49)
Chicken	Initial	53,691	10,380 (19)
	Improved	54,102	11,039 (20)
Rice	Initial	37,802	8,697 (23)
	Improved	36,996	7,540 (20)
Arabidopsis	Initial	50,101	33,297 (66)
	Improved	44,912	32,148 (71)

Percentages are shown in paranthesis

Table S3: No. of iterations taken by ROAST and the numbers of contigs in which different types of errors were identified and fixed by ROAST for each dataset

Species	# of iterati-ons	Incomplete super-transcript	Fragmented super-transcript	Local mis-assemblies				
				False chim-eras	Missing sequences	Unsuppor-ted insertions	Transl-ocations	Inve-rsion
**Human**	27	38,210	6,069	2,460	711	2,268	324	24
**Mouse**	39	21,643	2,745	1,798	1,670	901	49	10
**Chicken**	28	48,647	5,383	4,063	642	3,215	847	25
**Rice**	20	32,197	3,067	1,764	339	2,061	109	11
**Arabidopsis**	19	17,145	2,202	4,535	589	2,897	212	77
